# A concept for international societally relevant microbiology education and microbiology knowledge promulgation in society

**DOI:** 10.1111/1751-7915.14456

**Published:** 2024-05-27

**Authors:** Kenneth Timmis, John E. Hallsworth, Terry J. McGenity, Rachel Armstrong, María Francisca Colom, Zeynep Ceren Karahan, Max Chavarría, Patricia Bernal, Eric S. Boyd, Juan Luis Ramos, Martin Kaltenpoth, Carla Pruzzo, Gerard Clarke, Purificación López‐Garcia, Michail M. Yakimov, Jessamyn Perlmutter, Chris Greening, Emiley Eloe‐Fadrosh, Willy Verstraete, Olga C. Nunes, Oleg Kotsyurbenko, Pablo Iván Nikel, Paola Scavone, Max M. Häggblom, Rob Lavigne, Frédérique Le Roux, James K. Timmis, Victor Parro, Carmen Michán, José Luis García, Arturo Casadevall, Shelley M. Payne, Joachim Frey, Omry Koren, James I. Prosser, Leo Lahti, Rup Lal, Shailly Anand, Utkarsh Sood, Pierre Offre, Casey C. Bryce, Allen Y. Mswaka, Jörg Jores, Betül Kaçar, Lars Mathias Blank, Nicole Maaßen, Phillip B. Pope, Horia L. Banciu, Judith Armitage, Sang Yup Lee, Fengping Wang, Thulani P. Makhalanyane, Jack A. Gilbert, Thomas K. Wood, Branka Vasiljevic, Mario Soberón, Zulema Udaondo, Fernando Rojo, Jyoti Prakash Tamang, Tatiana Giraud, Jeanne Ropars, Thaddeus Ezeji, Volker Müller, Hirofume Danbara, Beate Averhoff, Angela Sessitsch, Laila Pamela Partida‐Martínez, Wei Huang, Søren Molin, Pilar Junier, Ricardo Amils, Xiao‐Lei Wu, Eliora Ron, Huseyin Erten, Elaine Cristina Pereira de Martinis, Alexander Rapoport, Maarja Öpik, W. Donald R. Pokatong, Courtney Stairs, Mohammad Ali Amoozegar, Jéssica Gil Serna

**Affiliations:** ^1^ Institute for Microbiology Technical University of Braunschweig Braunschweig Germany; ^2^ Institute for Global Food Security Queens University Belfast Belfast UK; ^3^ School of Life Sciences University of Essex Colchester UK; ^4^ Department of Architecture KU Leuven Gent Belgium; ^5^ Laboratory of Medical Mycology Universidad Miguel Hernández Alicante Spain; ^6^ Department of Medical Microbiology Ankara University School of Medicine Ankara Turkey; ^7^ Escuela de Química, CIPRONA Universidad de Costa Rica & Centro Nacional de Innovaciones Biotecnológicas (CENIBiot) San José Costa Rica; ^8^ Department of Microbiology Universidad de Sevilla Sevilla Spain; ^9^ Department of Microbiology and Cell Biology Montana State University Bozeman Montana USA; ^10^ Consejo Superior de Investigaciones Cientificas Estación Experimental del Zaidín Granada Spain; ^11^ Department of Insect Symbiosis Max Planck Institute for Chemical Ecology Jena Germany; ^12^ Department of Earth, Environmental and Life Sciences (DISTAV) University of Genoa Genoa Italy; ^13^ Department of Psychiatry and Neurobehavioural Science and APC Microbiome Ireland University College Cork Cork Ireland; ^14^ Ecologie Systématique Evolution, CNRS Université Paris‐Saclay Gif‐sur‐Yvette France; ^15^ Institute of Polar Sciences Italian National Research Council (ISP‐CNR) Messina Italy; ^16^ Department of Molecular Biosciences University of Kansas Laurence Kansas USA; ^17^ Department of Microbiology, Biomedicine Discovery Institute Monash University Clayton Australia; ^18^ Metagenome Program, DOE Joint Genome Institute Lawrence Berkeley National Lab Berkeley California USA; ^19^ Center for Microbial Ecology and Technology (CMET) Ghent University Ghent Belgium; ^20^ LEPABE‐Laboratory for Process Engineering, Environment, Biotechnology and Energy, Faculty of Engineering University of Porto Porto Portugal; ^21^ Higher School of Ecology Yugra State University Khanty‐Mansiysk Russia; ^22^ Systems Environmental Microbiology Group, The Novo Nordisk Foundation Center for Biosustainability Technical University of Denmark Lyngby Denmark; ^23^ Departamento de Microbiología Instituto de Investigaciones Biológicas Clemente Estable Montevideo Uruguay; ^24^ Department of Biochemistry and Microbiology Rutgers University New Brunswick New Jersey USA; ^25^ Laboratory of Gene Technology KU Leuven Heverlee Belgium; ^26^ Département de Microbiologie, Infectiologie et Immunologie Université de Montréal Montreal Quebec Canada; ^27^ Department of Political Science University of Freiburg Freiburg im Breisgau Germany; ^28^ Centro de Astrobiología (CAB) CSICINTA Madrid Spain; ^29^ Departamento de Bioquímica y Biología Molecular Universidad de Córdoba Córdoba Spain; ^30^ Environmental Biotechnology Laboratory Centro de Investigaciones Biológicas Margarita Salas (CIB‐MS, CSIC) Madrid Spain; ^31^ Department of Molecular Microbiology and Immunology Johns Hopkins Bloomberg School of Public Health Baltimore Maryland USA; ^32^ Department of Molecular Biosciences University of Texas at Austin Austin Texas USA; ^33^ Vetsuisse Faculty University of Bern Bern Switzerland; ^34^ Azrieli Faculty of Medicine Bar‐Ilan University Safed Israel; ^35^ School of Biological Sciences University of Aberdeen Aberdeen UK; ^36^ Department of Computing University of Turku Turku Finland; ^37^ Acharya Narendra Dev College University of Delhi New Delhi Delhi India; ^38^ Department of Zoology, Deen Dayal Upadhyaya College University of Delhi New Delhi Delhi India; ^39^ Department of Zoology, Kirori Mal College University of Delhi New Delhi Delhi India; ^40^ Department of Marine Microbiology and Biogeochemistry NIOZ–Royal Netherlands Institute for Sea Research Den Burg The Netherlands; ^41^ Cabot Institute for the Environment University of Bristol Bristol UK; ^42^ University of Nottingham Nottingham UK; ^43^ Institute of Veterinary Bacteriology University of Bern Bern Switzerland; ^44^ Department of Bacteriology University of Wisconsin–Madison Madison Wisconsin USA; ^45^ Institute of Applied Microbiology RWTH Aachen University Aachen Germany; ^46^ Faculty of Biosciences Norwegian University of Life Sciences As Norway; ^47^ Faculty of Chemistry, Biotechnology and Food Science NMBU As Norway; ^48^ Department of Molecular Biology and Biotechnology Babeș‐Bolyai University Cluj‐Napoca Romania; ^49^ Department of Biochemistry University of Oxford Oxford UK; ^50^ Department of Chemical & Biomolecular Engineering KAIST (Korea Advanced Institute of Science and Technology) Daejeon South Korea; ^51^ International Center for Deep Life Investigation (ICDLI) Shanghai JiaoTong University Shanghai China; ^52^ Department of Biochemistry, Genetics and Microbiology University of Pretoria Hatfield South Africa; ^53^ Department of Pediatrics and Scripps, Institution of Oceanography UC San Diego La Jolla California USA; ^54^ Department of Chemical Engineering Pennsylvania State University University Park Pennsylvania USA; ^55^ Institute of Molecular Genetics and Genetic Engineering University of Belgrade Belgrade Serbia; ^56^ Instituto de Biotecnología Universidad Nacional Autónoma de México Mexico City Mexico; ^57^ Department of Microbial Biotechnology, Centro Nacional de Biotecnología CSIC Madrid Spain; ^58^ Department of Microbiology Sikkim University Gangtok Sikkim India; ^59^ Laboratoire Ecologie, Systématique et Evolution (ESE) Université Paris‐Saclay Gif‐sur‐Yvette France; ^60^ Department of Animal Sciences The Ohio State University & OARDC Wooster Ohio USA; ^61^ Molekulare Mikrobiologie & Bioenergetik Goethe‐Universität Frankfurt Frankfurt Germany; ^62^ Shibasaburo Kitasato Memorial Museum Kitasato University Minato‐ku Japan; ^63^ AIT Austrian Institute of Technology Tulln Austria; ^64^ Departamento de Ingeniería Genética Centro de Investigación y de Estudios Avanzados‐Unidad Irapuato Irapuato Mexico; ^65^ Department of Engineering Science University of Oxford Oxford UK; ^66^ DTU Biosustain Lyngby Denmark; ^67^ Laboratory of Microbiology University of Neuchâtel Neuchâtel Switzerland; ^68^ Centro de Biología Molecular Severo Ochoa Madrid Spain; ^69^ Department of Energy Resources Engineering Peking University Beijing China; ^70^ The Shmunis School of Biomedicine and Cancer Research Tel Aviv University Tel Aviv Israel; ^71^ Department of Food Engineering Cukurova University Adana Turkey; ^72^ Faculdade de Ciências Farmacêuticas de Ribeirão Preto Universidade de São Paulo São Paulo Brazil; ^73^ Institute of Microbiology and Biotechnology University of Latvia Riga Latvia; ^74^ Department of Botany University of Tartu Tartu Estonia; ^75^ Department of Food Technology Universitas Pelita Harapan Tangerang Indonesia; ^76^ Department of Biology Lund University Lund Sweden; ^77^ Department of Microbiology University of Tehran Tehran Iran; ^78^ Departamento de Genética, Fisiología y Microbiología Universidad Complutense de Madrid Madrid Spain

**Keywords:** critical‐systems thinking, curriculum change, democratisation of microbiology knowledge, global citizenship, International Microbiology Literacy Initiative (IMiLI), lifelong learning, microbial technologies, societal inequalities, sustainability‐sustainable development goals

## Abstract

**Executive summary:**

Microbes are all pervasive in their distribution and influence on the functioning and well‐being of humans, life in general and the planet. Microbially‐based technologies contribute hugely to the supply of important goods and services we depend upon, such as the provision of food, medicines and clean water. They also offer mechanisms and strategies to mitigate and solve a wide range of problems and crises facing humanity at all levels, including those encapsulated in the sustainable development goals (SDGs) formulated by the United Nations. For example, microbial technologies can contribute in multiple ways to decarbonisation and hence confronting global warming, provide sanitation and clean water to the billions of people lacking them, improve soil fertility and hence food production and develop vaccines and other medicines to reduce and in some cases eliminate deadly infections. They are the foundation of biotechnology, an increasingly important and growing business sector and source of employment, and the centre of the *bioeconomy*, *Green Deal*, etc. But, because microbes are largely invisible, they are not familiar to most people, so opportunities they offer to effectively prevent and solve problems are often missed by decision‐makers, with the negative consequences this entrains. To correct this lack of vital knowledge, the International Microbiology Literacy Initiative–the IMiLI–is recruiting from the global microbiology community and making freely available, teaching resources for a curriculum in societally relevant microbiology that can be used at all levels of learning. Its goal is the development of a society that is literate in relevant microbiology and, as a consequence, able to take full advantage of the potential of microbes and minimise the consequences of their negative activities. In addition to teaching about microbes, almost every lesson discusses the influence they have on sustainability and the SDGs and their ability to solve pressing problems of societal inequalities. The curriculum thus teaches about sustainability, societal needs and global citizenship. The lessons also reveal the impacts microbes and their activities have on our daily lives at the personal, family, community, national and global levels and their relevance for decisions at all levels. And, because effective, evidence‐based decisions require not only relevant information but also critical and systems thinking, the resources also teach about these key generic aspects of deliberation.

The IMiLI teaching resources are learner‐centric, not academic microbiology‐centric and deal with the microbiology of everyday issues. These span topics as diverse as owning and caring for a companion animal, the vast range of everyday foods that are produced via microbial processes, impressive geological formations created by microbes, childhood illnesses and how they are managed and how to reduce waste and pollution. They also leverage the exceptional excitement of exploration and discovery that typifies much progress in microbiology to capture the interest, inspire and motivate educators and learners alike.

The IMiLI is establishing Regional Centres to translate the teaching resources into regional languages and adapt them to regional cultures, and to promote their use and assist educators employing them. Two of these are now operational. The Regional Centres constitute the interface between resource creators and educators–learners. As such, they will collect and analyse feedback from the end‐users and transmit this to the resource creators so that teaching materials can be improved and refined, and new resources added in response to demand: educators and learners will thereby be directly involved in evolution of the teaching resources. The interactions between educators–learners and resource creators mediated by the Regional Centres will establish dynamic and synergistic relationships–a global societally relevant microbiology education ecosystem–in which creators also become learners, teaching resources are optimised and all players/stakeholders are empowered and their motivation increased.

The IMiLI concept thus embraces the principle of teaching societally relevant microbiology embedded in the wider context of societal, biosphere and planetary needs, inequalities, the range of crises that confront us and the need for improved decisioning, which should ultimately lead to better citizenship and a humanity that is more sustainable and resilient.

**Abstract:**

The biosphere of planet Earth is a microbial world: a vast reactor of countless microbially driven chemical transformations and energy transfers that push and pull many planetary geochemical processes, including the cycling of the elements of life, mitigate or amplify climate change (e.g., Nature Reviews Microbiology, 2019, 17, 569) and impact the well‐being and activities of all organisms, including humans. Microbes are both our ancestors and creators of the planetary chemistry that allowed us to evolve (e.g., Life's engines: How microbes made earth habitable, 2023). To understand how the biosphere functions, how humans can influence its development and live more sustainably with the other organisms sharing it, we need to understand the microbes. In a recent editorial (Environmental Microbiology, 2019, 21, 1513), we advocated for improved microbiology literacy in society. Our concept of microbiology literacy is not based on knowledge of the academic subject of microbiology, with its multitude of component topics, plus the growing number of additional topics from other disciplines that become vitally important elements of current microbiology. Rather it is focused on microbial activities that impact us–individuals/communities/nations/the human world–and the biosphere and that are key to reaching informed decisions on a multitude of issues that regularly confront us, ranging from personal issues to crises of global importance. In other words, it is knowledge and understanding essential for adulthood and the transition to it, knowledge and understanding that must be acquired early in life in school. The 2019 Editorial marked the launch of the International Microbiology Literacy Initiative, the IMiLI.

**Here, we present:**

- our concept of how microbiology literacy may be achieved and the rationale underpinning it;
- the type of teaching resources being created to realise the concept and the framing of microbial activities treated in these resources in the context of sustainability, societal needs and responsibilities and decision‐making; and
- the key role of Regional Centres that will translate the teaching resources into local languages, adapt them according to local cultural needs, interface with regional educators and develop and serve as hubs of microbiology literacy education networks.

The topics featuring in teaching resources are learner‐centric and have been selected for their inherent relevance, interest and ability to excite and engage. Importantly, the resources coherently integrate and emphasise the overarching issues of sustainability, stewardship and critical thinking and the pervasive interdependencies of processes. More broadly, the concept emphasises how the multifarious applications of microbial activities can be leveraged to promote human/animal, plant, environmental and planetary health, improve social equity, alleviate humanitarian deficits and causes of conflicts among peoples and increase understanding between peoples (Microbial Biotechnology, 2023, 16(6), 1091–1111). Importantly, although the primary target of the freely available (CC BY‐NC 4.0) IMiLI teaching resources is schoolchildren and their educators, they and the teaching philosophy are intended for all ages, abilities and cultural spectra of learners worldwide: in university education, lifelong learning, curiosity‐driven, web‐based knowledge acquisition and public outreach. The IMiLI teaching resources aim to promote development of a global microbiology education ecosystem that democratises microbiology knowledge.

## Graphical abstract

Microbes are all pervasive in their distribution and influence on the functioning and wellbeing of humans, life in general and the planet. Understanding their activities is vital for decisions and policies at all levels, in particular those relating to sustainability. The International Microbiology Literacy Initiative (IMiLI) is a global microbiology community creating freely available teaching resources to promote literacy in societally‐relevant microbiology worldwide. The resources also teach about global citizenship, sustainability, and critical and systems thinking. IMiLI Regional Centres adapt and translate resources to regional needs and languages, and promote their use in educational facilities and web‐based learning.

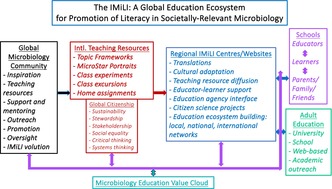

## INTRODUCTION

### The need for change in educational curricula

The purpose of education (see Martin Luther King: http://okra.stanford.edu/transcription/document_images/Vol01Scans/123_Jan‐Feb1947_The%20Purpose%20of%20Education.pdf; and Biesta, 2008 for discussions on why this is important) is to develop the minds and abilities of children, to help them realise their full individual potentials and to provide them with the knowledge and understanding they need to flourish as adults and manage the challenges and opportunities they will face that will require or benefit from informed decisions and actions. Education also needs to promote character building, instil moral and ethical values and encourage development of a sense of collective responsibility/citizenship (https://erpapers.columbian.gwu.edu/good‐citizenship‐purpose‐education) for social justice and sustainability (e.g., https://campaignforeducation.org/en/take‐action/transformative‐education).

School curricula of different cultures have evolved to provide fundamental knowledge and skills considered to be key to the needs and responsibilities of adults of those cultures. However, education is not only for children. For various reasons, many children do not receive adequate education but may be able to catch up as adults. Other adults extend their childhood education by exploring learning opportunities throughout life to gain new employment‐relevant skill sets or for mental stimulation and/or personal satisfaction (https://www.uil.unesco.org/en/unesco‐institute/mandate/lifelong‐learning).

The world of 2024 is very different from that of earlier decades, is changing rapidly and is compelling the adoption of new approaches, solutions and behaviours. The children of 2024 and their educational needs are also different from those of earlier generations and require new educational content to create new understanding, abilities, skill sets and mindsets that will endow future adult populations with greater fitness and resilience for the world they will inherit (e.g., Zhang et al., 2022).

Educators continuously make efforts to adapt their teaching to these changing conditions, but there is still a growing sense of the need for a reset of current school curricula to better align with current and future needs (e.g., https://widgets.weforum.org/nve‐2015/chapter1.html; https://www.un.org/sites/un2.un.org/files/report_on_the_2022_transforming_education_summit.pdf). Notwithstanding these efforts and strategic views, educational curricula are generally rather rigid and evolve too slowly to keep up with societal needs and new innovations; in this sense, the current mechanism of evolution of the content of educational curricula is inadequate and unsustainable. There is a need to incorporate new topics into educational curricula and to evolve curricula that are more flexible and responsive to societal requirements.

We present here a new paradigm for incorporating a subject that we consider to be vital for education, namely societally relevant microbiology. We also illustrate how it can be responsive to newly developing needs and hence serve as a model to create sustainable, evolvable curricula.

### The need to incorporate societally relevant microbiology in educational curricula

Microbes were the first and only forms of life for the first 3 billion years of evolution (e.g., Bertrand, 2015; Knoll, 2015; Mahendrarajah et al., 2023). During this time, they shaped planetary surface chemistry in such a way that complex life forms could develop, evolve and diversify into the current members of the biosphere (Sousa et al., 2013; Ehrlich et al., 2015; Jelen et al., 2016; Moore et al., 2017; Raanan et al., 2018; Boyd et al., 2023; Falkowski, 2023; Goldman & Kaçar, 2023; López‐García & Moreira, 2023). Microbes colonise and pervasively influence all life forms, cycle elements and hence wastes of all organisms, including those generated by human activities (agricultural, industrial, etc.), interchange and exchange diverse forms of food, nutrients and energy, produce a substantial fraction of the oxygen used in the biosphere (Glibert & Mitra, 2022), and considerably determine human and planetary well‐being (Falkowski et al., 2008; Flandroy et al., 2018; Hou et al., 2022; Ogunrinola et al., 2020; Schmidt et al., 2019; Sessitsch et al., 2023). Microbes are central to all food webs, either as primary producers, fixing carbon dioxide coupled with harvesting solar or chemical energy or by using organic materials, such as exudates, waste products or dead cells and organisms. Their small size, and thus large surface area to volume ratio, together with their abundance and metabolic diversity, combine to make microbes the engine that drives global biogeochemical cycles (Cavicchioli et al., 2019; Glibert & Mitra, 2022).

Humans often regard themselves as at the apex of food chains/webs. But microbial pathogens can kill all organisms of food webs, including humans and other microbes live off their dead bodies. So, microbes collectively may justifiably be considered to be at the apex as well as the base of food chains/webs. As a consequence, they prevent the dominance of otherwise successful species and so play a major role in maintaining biodiversity (Altizer et al., 2003; Bever et al., 2015; Ribeirinho‐Soares et al., 2022). (A key distorting influence in biodiversity maintenance, is of course the human who, through advances in medicine, has been able to reduce infection‐caused mortality and thereby thwarted this natural ecological activity, and has uncoupled normal population dynamics from resource availability and thereby directly caused an enormous loss of biodiversity (Cardinale et al., 2012; Langdon et al., 2016; Chase et al., 2020; Mougi, 2022; Dornelas et al., 2023; Selaković et al., n.d.). Microbes and their activities are therefore pivotal to the functioning of the biosphere. This is a relatively recent realisation, which probably explains why, despite its importance, microbiology education is less advanced than the level that is essential for societal needs.

**The world of 2024 and its educational needs are different from those of yesteryear**

- Human population growth and increasing life spans, which are exacerbating shortages of natural resources, prejudicing human and biosphere sustainability and exacerbating social inequalities (e.g., see Merz et al., 2023; Timmis & Hallsworth, 2022)
- Global crises facing humanity, e.g., global warming, antimicrobial resistance
- Exponential increase in data and new means to analyse it (via artificial intelligence)
- Explosion of knowledge, with its consequences for human needs and endeavours
- Global hyper‐connectivity–physical, but especially digital–with increased access to information and mis‐ and disinformation (www.unhcr.org/innovation/wp‐content/uploads/2022/02/factsheet‐4.pdf) and a need to distinguish trusted from nefarious sources
- A need for an intimate knowledge of nature (e.g., Pascual et al., 2023 and citations therein),
- The demand for education to promote the ability to reason and form objective decisions
- Societal fragmentation
- Increasing requirement for education to deal with societal issues and their potential solutions
- The obligation for education to deal with sustainability and its requirements, e.g., net‐zero carbon, a circular economy and frugal innovation
- Increasing inequality in education, especially among children
- Increasing recognition of diversity of learning needs
- Increasing recognition of the importance of lifelong learning

We previously made the case for microbiology literacy (Timmis et al., 2019; for a definition of literacy, see: https://uis.unesco.org/en/glossary‐term/literacy), outlining the pervasive impacts of the microbial world on a wide spectrum of issues that hugely affect humanity. And, as can be appreciated from Data S1, microbial activities regularly affect us all personally, as individuals. Influencing these activities requires that we understand them. Knowledge of relevant aspects of microbiology is key to understanding life itself and our place in the biosphere, optimising our well‐being and that of other organisms and hence to biosphere sustainability.

**Why the society needs to become microbiology literate: understanding and managing the manifold impacts of microbes on humanity and the biosphere**

- *Planetary importance*: microbes are the great chemists and transformers (the planetary cyclers and recyclers), creating‐transforming‐responding to a vast array of chemicals that influence and regulate the biosphere in many ways.
- *Sustainability importance*: the developmental trajectory of humanity has long been unsustainable. Microbial processes are crucial to efforts to achieve the sustainable development goals (SDGs) (https://sdgs.un.org/2030agenda; Timmis & Timmis, 2017; see also https://ami‐journals.onlinelibrary.wiley.com/toc/17517915/2017/10/5)
- *Crisis importance*: microbes are at the centre of multiple, intertwined, *existential* crises, both as mitigating agents/solutions and causes, including global warming and climate tipping points, planetary boundaries, environmental pollution, desertification/soil fertility‐health/agricultural productivity and humanitarian polycrises associated both with these issues and with prejudicial human activities (some of which are encapsulated in the wise quote of Mahatma Gandhi: ‘Earth provides enough to satisfy every man's needs, but not every man's greed.’), including poverty, hunger, lack of sanitation and clean water, antimicrobial resistance, resurgence of almost eradicated infectious diseases, pandemics of new diseases and inadequate access to healthcare.
- *Socio‐economic importance*: microbes provide/inspire new therapies, vaccines, diagnostics, chemicals, materials, foods, food supplements, fermented foods, flavourings, enable production of biofuels from renewable sources and wastes, increased crop yields, environmentally friendly recovery of natural resources, waste management–recycling–bioremediation, even human dwellings (see Armstrong, 2023), are at the core of the Bioeconomy/Green Deal/One Health, etc. Microbial technologies can provide key basic goods and services in resource‐poor settings and thereby eliminate deficits and reduce asymmetries of such services among peoples (Anand et al., 2023).
- *Personal/individual importance*: microbes are part of us and all other organisms of the biosphere and provide essential services and regulate to a significant extent our physical and mental well‐being.

It is not only a question of understanding how microbes affect us but also appreciating how we affect them conciously and unwittingly. Since microbial activities are existential for the proper functioning of vital biosphere and planetary processes (e.g., see Cavicchioli et al., 2019; https://octogroup.org/news/tiny‐mighty‐ocean‐health‐depends‐bacteria‐and‐viruses/), it is equally important that our decisions do not prejudice (harm or eliminate) key microbial systems and their activities or functions (see also: https://associationofanaesthetists‐publications.onlinelibrary.wiley.com/doi/10.1111/anae.16168). To recall the poignant and prophetic insight of Curtis (2006): ‘For if the last blue whale choked to death on the last panda, it would be disastrous but not the end of the world. But if we accidentally poisoned the last two species of ammonia oxidizers, that would be another matter. It could be happening now and we wouldn't even know…’ Such microbial activities are foundational to clean drinking water and the health of aquatic ecosystems on which we humans critically depend. Microbes are essential to us and, to maintain and extend their vital support, we need to protect them and their habitats (e.g., see also https://www.microbiotavault.org).

The International Microbiology Literacy Initiative was founded to enable and promote microbiology literacy to benefit both humanity and the planet. *Its explicit aim is to realise the teaching of societally relevant microbiology in every school and educational venue worldwide able and willing to embrace it*. The IMiLI aims not only to create awareness in children of how microbes touch their lives in myriad ways with so many important consequences, but also to reveal a wide range of personal activity‐/experience‐/interest‐relevant microbiology topics and thereby enrich their education, provide relevant life skills and support their decision‐making as adults. It also strives to promote consideration of such everyday decisions in wider contexts–of related issues, of regional and global issues, of sustainability and Grand Challenges–and to foster critical and systems analysis of relevant parameters in decision‐making and development of a sense of responsibility for decisions taken.

Efforts to improve microbiology literacy in society must also extend to adults. In this regard, the IMiLI teaching resources can be used in formal teaching of adults in schools (lifelong learning; https://www.uil.unesco.org/en/unesco‐institute/mandate/lifelong‐learning) and universities and in informal, web‐based learning by both adults and children. Some resources, such as family projects, are being created specifically to propagate knowledge within childrens’ family–friend networks.

In this article, we discuss:

- the IMiLI education concept and rationale,
- the teaching resources being created by hundreds of microbiologists worldwide,
- how the resources incorporate generic issues of sustainability, critical and systems thinking and problem‐solving, stewardship–stakeholder–civic responsibilities, the interconnectedness of issues and processes and societal inequalities,
- the roles of IMiLI Regional Centres, the key interfaces between teaching resource creators and learners and
- a mechanism for the dynamic and timely evolution of teaching resources through educator–student feedback to resource creators.

## CONSIDERATIONS THAT FRAMED THE DEVELOPMENT OF THE IMiLI CONCEPT

While the creation of new school curricula that integrate the issues listed above constitutes a challenge, it also presents an opportunity to incorporate some additional educational parameters that are broadly important or timely. We have therefore considered several additional criteria to steer development of the microbiology curriculum concept and teaching resources. As might be expected, some of these are intertwined and interdependent.

### Relevance

The aim of the IMiLI is not to create microbiologists with a curriculum built around academic microbiology, but rather to reveal microbial contributions to child‐/society‐relevant issues (e.g., McGenity et al., 2020). It also aims to describe microbial activities and properties that children (and adults) will find interesting and exciting, to leverage their insatiable thirst for knowledge of fascinating themes while reinforcing the importance of microbes and increasing the pleasure of learning. Emphasis on relevance will stimulate the all‐important 3 E's: *Excitement, Engagement* and *Empowerment* (Timmis, 2023). The 3 E's should awaken and sustain fascination for microbes and their activities, so that children become self‐motivating and explore according to their interests. Topics taught should foster the spirit of adventure, be discovery‐centric and, where possible, synergise with existing interests, such as astrobiology to leverage prevalent excitement about space exploration and science fiction such as the Star Wars and Star Trek franchises (see also Noor, 2018; https://www.youtube.com/watch?v=‐_y20wLlm‐k).

### Motivation

Motivation is key to an engaged, enjoyable and effective education and is particularly important in educators whose enthusiasm plays a central role in creating and sustaining pupil interest. While the 3 E's are motivators for pupils and educators alike, the contents and formats of the teaching materials must kindle the natural passion of educators. Immersive activities that pupils and educators can enjoy together and even co‐create, such as class experiments, excursions, competitions, citizen science projects, can provide additional motivation (the 3 I's: *immersion, imagination* and *inspiration*; see also McGenity et al., 2020). *Empowerment*, as alluded to below, is also a motivating factor as it provides a sense of ownership and de facto perspective for shaping the further development of class projects.

### Engagement with microbes

One of the key pedagogical handicaps in microbiology education is that most microbes cannot be directly seen as  study objects. (That said, invisibility can also trigger curiosity and imagination since, for some children, this can also make them mysterious and especially fascinating and their discovery especially rewarding). Rendering microbes and their processes visible and creating corresponding mental images that automatically become associated with topic narratives, is the challenge. Teaching materials are therefore image‐rich, narratively engaging and will be supported by visualisation‐engagement‐immersion resources.

**Engagement with microbes**

- Galleries of portraits of the most famous, fascinating and extravagant microbes
- Lesson‐specific Class Experiments (e.g., making yoghurt, soy sauce, fermented cabbage and other vegetables, bread, pizza), hands‐on activities that can get children in direct contact with the microbes that impact them
- Recommendations for teacher‐/expert‐accompanied class excursions designed to reveal to children (and, by extension, their families and friends) diverse examples of microbiology in action in their immediate localities, so that they mentally connect everyday observations and experiences with the underlying microbes and their activities (McGenity et al., 2020)
- Lesson‐specific Multimedia Teaching Aids (MTAs), including images, cartoons, comics, videos, video games
- Custom videos of field trips/sampling campaigns to expose the thrill of discovery of microbiology research in exotic locations (e.g., see https://www.youtube.com/watch?v=CQxqmA3CTjU)

### Countering germophobia

Although Louis Pasteur stated *life would not long remain possible in the absence of microbes*, societal knowledge of microbes is largely restricted to infections, disease and deterioration of food and materials, so microbes have a bad name, *germs*, with its corresponding connotation and are generally considered to be dangerous, disgusting–something to be feared (see, e.g., https://lyrics.lyricfind.com/en‐GB/lyrics/dionne‐warwick‐ill‐never‐fall‐in‐love‐again‐1; https://www.youtube.com/watch?v=oN4Bkal4eFU). This perception has been amplified by incessant commercial advertising of germicidal products for cleaning either the home or human body surfaces and by the–at times emotionally supercharged–gene technology debate (de Lorenzo, 2010). Society is largely germophobic, with the consequence that unnaturally restricted exposure to microbes negatively impacts the development of healthy immune systems of children (Mulder et al., 2011; Okada et al., 2010; Rook, 2023).

But around 50% of the cells of the human body are microbes (Sender et al., 2016), with each human being composed of communities of diverse types of (differentiated) human cells and (phylogenetically diverse) microbial cells. The human organism nevertheless functions not as two independently acting entities, but rather as a coherent and mostly coordinated whole–a metaorganism/holobiont–with the microbial cells, the microbiome, providing all manner of essential goods and services to the human cells and the human providing nutrients and habitats for the microbiome (Dahl et al., 2020; de Vos et al., 2022; O'Toole & Max Paoli, 2023; Postler & Ghosh, 2017; Utter et al., 2020; van de Guchte et al., 2018). *Microbe demonisation is self‐demonisation!*

Germophobia is a huge societal bias–a form of socio‐cultural disease–that hinders balanced consideration of microbial activities and the process of arriving at informed objective opinions, policies and decisions relating to issues impacted by these activities. Crucially, it hinders us from reaping their enormous potential benefits. The combination of germophobia, germicidal product use (which has been amplified by the COVID‐19 pandemic) and inappropriate antibiotic use in humans and food production, has undoubtedly altered human/animal/environmental microbiome community compositions and their interactions, with unforeseen ecological consequences (Cabello et al., 2013; Coque et al., 2023; Grenni et al., 2018; Kraemer et al., 2019; Larsson & Flach, 2022).

Efforts must be made to counter germophobia by presenting a balanced picture of desirable/positive and undesirable activities of microbes, how we ourselves evolved from them, how our own human microbiome protects us and, in taking care of our microbiome, we take care of ourselves and how they and their engineered relatives can be exploited to produce a range of products and services of enormous benefit to humanity. *Crucially, we need to explain the negative consequences of ignoring and not exploiting and safeguarding the beneficial microbes and their activities*. We need to encourage people to follow the path of ‘Positive Microbiology' (Oli, 2023).

### Worldwide online accessibility

Teaching resources tend to be restricted in accessibility, for example, because of being created in only one or a few languages, designed for a particular education system/culture, or produced commercially and being too costly for some schools. A fundamental principle of the IMiLI is that its teaching resources will be made available online without cost (CC BY‐NC 4.0). Initially, the resources will be made available in English, but then, as IMiLI Regional Centres (see below) become progressively established, in all major languages, translated and adapted to local needs and cultures. Thus, the IMiLI aims to create the teaching resources for a global curriculum of societally relevant microbiology knowledge, freely accessible worldwide by educators and pupils (and adults with for interest in acquiring or disseminating this knowledge) in their own language (see also unesco.org/en/legal‐affairs/recommendation‐open‐educational‐resources‐oer).

### Versatility: Applicable to diverse educational settings defined by different cultures/priorities/education systems

The strategy adopted was to create stand‐alone, modular teaching materials that can be used independently, with no imperative to cover a specific spectrum of issues or to follow a pre‐determined topic sequence: educators/schools/universities/education authorities can select the topics appropriate to their system, culture, priorities and desired learning outcomes and chart their own learning paths. The fact that the teaching resources have been contributed by the global community of microbiologists, representing diverse cultures and using a common and universal terminology, should facilitate adaptation of the resources to local and current needs.

### Sustainability and microbiology education

For humanity to continue to survive on planet Earth, it must adopt sustainable lifestyles and practices (see, e.g., Timmis & Hallsworth, 2022). In an attempt to re‐orient the developmental trajectory of humans towards greater sustainability, the United Nations formulated its Sustainable Development Goals (https://sdgs.un.org/2030agenda). However, for the SDGs to fulfil their purpose, they must be achieved by 2030. Interim assessment of progress indicates that this is no longer possible (https://news.un.org/en/story/2023/07/1138777; Bexell & Jönsson, 2016).

Sustainable development and effectively confronting some of the major humanitarian and planetary challenges will require fundamental changes in the way things are done, financed and regulated (https://international‐review.icrc.org/articles/re‐evaluating‐ihl‐in‐a‐triple‐planetary‐crisis‐924; https://www.ifrc.org/sites/default/files/2022‐11/20221108_ClimateSmartFinance.pdf; https://www.msf.ch/en/media/4711; Barnard et al., 2021; Merz et al., 2023). Fundamental changes will not only require new policies but also societal acceptance of the policies. Acceptance will be greatly facilitated by an understanding of the underlying issues and their consequences; that is, by education of the public. While there are regular exhortations to accelerate progress towards the SDGs (https://unece.org/statistics/press/halfway‐2030‐unece‐report‐shows‐we‐must‐accelerate‐progress‐achieve‐sdgs‐region; https://sdg.iisd.org/commentary/guest‐articles/we‐need‐7‐years‐of‐accelerated‐transformative‐action‐to‐achieve‐sdgs/), until recently (e.g., see https://unstats.un.org/sdgs/report/2022/The‐Sustainable‐Development‐Goals‐Report‐2022.pdf, p.51) there has been little discussion or action about the role of education in promoting sustainability. Since microbes have an impact on all SDGs (e.g., Timmis, de Lorenzo, et al., 2017; Timmis, de Vos, et al., 2017; https://ami‐journals.onlinelibrary.wiley.com/toc/17517915/2017/10/5; D'Hondt et al., 2021), the IMiLI educational materials will explicitly relate learning topics to SDGs and thereby de facto play a significant role in sustainability education.

### Social responsibility–global citizenship: the 3 S's

Education is not simply the acquisition of knowledge and understanding and cognitive development (e.g., see https://www.structural‐learning.com/post/jean‐piagets‐theory‐of‐cognitive‐development‐and‐active‐classrooms): it should also create awareness of problems/opportunities, their origins/causes/rationales, the potential consequences of action and, most importantly, of inaction and explore opportunities to overcome such problems. Education should, where possible, engender a sense of individual and collective responsibility–*global citizenship* (UN SDG Report, 2022. P. 51: ‘More effort is needed to fully mainstream sustainable development and global citizenship in national education systems.’ https://unstats.un.org/sdgs/report/2022/The‐Sustainable‐Development‐Goals‐Report‐2022.pdf) and promote the development of willingness to become involved: the principle of stewardship and stakeholder responsibility. Societal and planetary challenges and the importance and consequences of personal decisions and behaviour for these challenges, human well‐being and welfare and the primordial need for peace and harmony among and between peoples (Barnard et al., 2021; Anand et al., 2023), need to be articulated.

**Social responsibility–global citizenship**

The IMiLI concept embodies the 3 *S*'s: education of young people about *S*tewardship (of their own lives, their families, their communities, their nations, the planet and the universe), *S*takeholder responsibilities and *S*ustainability. Children are almost always idealists and believe in their ability to change the world for the better, so education should cultivate their inherent sense of civic duty and provide guidance on how to be actively involved and take actions relating to their future (https://www.unesco.org/en/global‐citizenship‐peace‐education/need‐know). Emphasising the 3 *S*'s should also help counter a growing egotism and focus on self.

### Interdependence of processes, the wider context and systems thinking

Our current polycrises (https://www.weforum.org/agenda/2023/01/global‐risks‐report‐2023‐experts‐davos2023/?utm_source=sfmc&utm_medium=email&utm_campaign=2793318_AgendaWeekly13January2023&utm_term=&emailType=Agenda%20Weekly; Ripple et al., 2020; Barnard et al., 2021), such as widespread poverty, hunger, desertification, deforestation, human‐animal–plant epidemics and pandemics, reducing biodiversity, environmental pollution, armed conflicts and regional deficits in access to healthcare, clean water and employment opportunities, are important examples of the interconnected nature of processes and challenges. Education needs to reveal the pervasiveness of interdependencies, exemplified by One Health (https://www.avma.org/sites/default/files/resources/onehealth_final.pdf; https://www.fao.org/3/cc2289en/cc2289en.pdf; Flandroy et al., 2018; Sinclair, 2019) to counteract a fragmented perception/understanding and consideration of issues in isolation (*silo thinking*), and to promote the necessity of considering the whole picture and the potential influence of connected parameters when making decisions. *Education needs to promote systems thinking*.

### Critical thinking

Information is essential but not sufficient for rational and objective decision making. All individuals have biases, which accumulate with age and are subjected to endless streams of influencing, spin, groupthink, dogmatism and, crucially, mis‐ and disinformation (www.unhcr.org/innovation/wp‐content/uploads/2022/02/Factsheet‐4.pdf). To counteract biases, the curriculum should promote critical thinking and teach the importance of evidence‐based decisioning. This should counter the spread of conspiracy theories which, for example, figured prominently during the COVID‐19 pandemic of 2020–2021 and which, because of a deficit of relevant knowledge about microbes, were more readily accepted by many.

### Mindsets: widening vistas and opening minds to a greater range of contexts and options

To some extent, all children are raised in environments with a restricted outlook and diversity and range of opinions, discussion topics, values and culture(s) experienced. This is perfectly natural and beneficial because it creates cultural identity and so fosters a sense of belonging. Nevertheless, it has the disadvantages that the full range of potential solutions to problems is inapparent and, under certain circumstances, it can encourage rejection of unfamiliar values without due consideration and may even promote discrimination against other cultures. It is therefore important to stimulate the development of curiosity and open‐mindedness, widen the vistas of children by exposing them to issues they would not ordinarily experience and encourage networking of children from different cultures.

### Disadvantaged children

A considerable proportion of children in school are disadvantaged in some way or other, such as by having illnesses that cause absences or, due to learning difficulties, neurodivergence, poverty‐related inadequate nutrition, sub‐optimal or distracting home lives, or gender biases (e.g., SDG 4.5) and some risk to fall by the wayside because of it. While many educators make heroic efforts to prevent this, they need to be supported by teaching materials that have a high excitement–interest–fascination value which engage, stimulate and encourage all pupils, including those on the margins and that deal with issues that motivate them to explore further and want to get to school in the morning and discover something new.

The creation of new curricula presents an opportunity to incorporate features that help educators to reach all the unique minds in their classrooms and provide disadvantaged children with equitable access to education through provision of

- readily comprehended, coherent stories about child‐centric issues and materials of high excitement–interest–fascination value, which helps engage, stimulate and motivate all pupils,
- teaching materials that can be readily disseminated, studied and understood outside of the classroom setting, which are particularly important for children who miss formal sessions (see *Versatility* above),
- multimedia resources and instructions for hands‐on activities which will cater to multiple learning needs, styles and contexts and
- online video presentations of class lessons

### Confronting societal inequalities

Many of the challenges facing us now and in the future have a significant element of societal inequality. For example, global warming leads to rising sea levels, net loss and salination of fertile coastal lands that reduces agricultural capacity and increases hunger and food prices and reductions in living space that increase population migrations which affect mental and physical health, all of which will disproportionately affect resource‐poor countries and communities. Similarly, epidemics and pandemics like COVID‐19 have a greater impact on communities lacking adequate prevention and treatment measures (Boserup et al., 2020; Kirby, 2020; Timmis & Brüssow, 2020). Acquisition of an understanding of issues underlying social inequalities and asymmetries in basic goods and services, and of available mitigation measures centred on microbial technologies, are vital to the formulation of policies and taking of decisions aimed at progress towards a just and harmonious society (Anand et al., 2023).

### Adaptability–evolvability

The world and its educational demands are changing rapidly so it is essential to develop educational ecosystems that are able to adapt in a timely fashion to changing societal needs. The traditional system of building knowledge in a sequential manner, while rational and for some subjects the only option, creates rigidity. Generating modular teaching resources means that they can evolve with minimal constraint and maximal flexibility: topics that have become less relevant can be archived, more relevant ones can be expanded and new ones created and incorporated, without prejudice to overall curriculum concept, content and coherence.

### Democratisation of microbiology and evolution of teaching materials

As knowledge advances, it becomes more complex and technical, something only for specialists and the well‐educated, which can result in the public becoming disenfranchised and disengaged. This in turn can lead to the uncoupling of society from knowledge of vital importance for its own well‐being and hence from personal involvement in policy development and scrutiny (e.g., https://www.nber.org/system/files/working_papers/w28112/w28112.pdf). A poorly informed public is more prone to support bad decisions and accept and spread conspiracy theories. This trend needs to be reversed, by using understandable language; by teaching key aspects in school (and more broadly in society); by being inclusive, i.e., knowledge of vitally important subjects such as microbiology needs to be democratised; and, above all, by creating an educational ecosystem that maximises the engagement and motivation of learners and encourages their potential to diffuse the knowledge they acquire, i.e., to act as multipliers, such that *learners become educators* (Timmis et al., 2020).

Since formal education prepares children for adulthood, school curricula need to evolve with a changing society and world and its evolving challenges. This evolution needs to respect the criteria listed above, including child‐ and society‐relevance and interest. The different stakeholders–educators, learners, society–must therefore have both the responsibility and opportunity to influence in a timely fashion the evolution of topic content and the nature of the educational resources being developed. There needs to be democratisation of the subject matter taught. For this, education systems need to build feedback loops between the creators and users of teaching materials. Moreover, mechanisms are needed to ensure that feedback is properly appraised and incorporated in an even‐handed fashion.

Democratisation also demands accessibility. The free accessibility of IMiLI materials to everyone and their creation in easy‐to‐understand language, will produce a level playing field for microbiology teaching in educational ecosystems all over the world. While the emphasis here is democratisation of societally relevant microbiology knowledge, society would clearly benefit from the democratisation of other disciplines or sub‐disciplines. One mechanism is online communities, such as the Carpentries (carpentries.org), or “learning clubs” that are a popular learning mode for instance in computationally oriented fields (e.g. microbial bioinformatics; see Hagan et al., 2020). We therefore encourage others to follow suit and erase financial barriers to teaching content that is high‐quality, informative and relevant, including content that at present is only accessible in tertiary education.

**Some criteria that steered the development of the IMiLI Teaching resources**

- Relevance: revealing microbial roles in, impacts on, everyday experiences and decisions, socio‐economic issues and global problems
- Learner‐educator motivation: revealing the excitement of microbial activities
- Sensorial engagement
- Counteraction of germophobia
- Worldwide online accessibility
- Global applicability: adaptability to different education systems and cultures
- Sustainability of humanity and of the Earth's biosphere
- Global citizenship
- The wider context and interdependencies of processes
- Critical and systems thinking, problem‐solving
- Broadening mind sets and widening vistas
- Inclusivity for disadvantaged students
- Illumination of social inequalities and ways and means of levelling up
- Timely evolution due to inherent versatility
- Democratisation of microbiology and educator–learner involvement in evolution of teaching materials

## THE CONCEPT OF THE IMiLI Curriculum

We therefore set out to create a curriculum that would focus on child‐centric, exciting, societally relevant topics that tap into the fascination of discovery and reveal and leverage the wide range of microbial activities and processes that affect us on a daily basis (see Data S1). The relevance of sustainability, social equity, stewardship and stakeholder awareness are emphasised where appropriate, as well as the wider context and the interdependencies of things. A conscious effort to promote active learning (Bean & Melzer, 2021; McGenity et al., 2020; Theobald et al., 2020; Yannier et al., 2021), critical thinking and informed decision‐making lies at the heart of the curriculum's ethos. Children are actively encouraged to become multipliers and promoters of microbiology literacy by disseminating newly acquired knowledge they find exciting to family and friends (Timmis et al., 2020).

One decision taken at the outset was to create a single *generic* and modular curriculum that could be adapted worldwide by educators for all relevant age groups and teaching aims. Creating resources individually tailored to each age group and target class would have required greater initial effort, potential fragmentation and disconnects between materials created for different groups and even greater downstream investment during international implementation (e.g., language translations). A single curriculum has the significant value of coherence: educators can use the same basic materials with age progression in the school. Indeed educators can themselves be taught with the same materials they subsequently use to teach. On the other hand, while the main resources of the curriculum–the class lessons (Topic Frameworks (TFs)) and MicroStar Portrait Galleries (see below)–are in general adaptable to different age groups, some of the ancillary resources, like some of the class experiments, will necessarily be more age‐specific.

## TEACHING RESOURCES BEING CREATED FOR THE SOCIETALLY RELEVANT MICROBIOLOGY EDUCATION CURRICULUM

The teaching resources currently being created by the IMiLI are listed below. To be accessible to educators of virtually all knowledge levels, children and other interested parties, they are mostly expressed in non‐technical language for understandability by non‐experts (see Data S2 for Instructions to authors).

**Teaching resources being created by the IMiLI and their *purposes***

- Topic Frameworks (TFs)–the class lessons: the *curriculum*
- Portrait Galleries–microbial halls of fame featuring the principle actors in TFs: *familiarisation*
- Multimedia teaching aids (MTAs): *visualisation*
- TF‐specific class experiments: *engagement with microbes and their processes*
- Virtual participation in field work in exotic locations: *excitement of exploration and discovery*
- Class excursion recommendations: *discovering microbes in action at local sites*
- Home assignments, family/friends projects: *independent exploration of microbial topics; knowledge amplification and diffusion*

### Topic frameworks–the class lessons

A TF is a knowledge framework of an engaging phenomenon or aspect of microbiology that provides educators with the principal information elements they need to teach their classes. The TF series is the core of the teaching resources. The TFs completed (200+) or in revision (100+) in April, 2024 and those in the pipeline or still to be recruited (100+), cover a wide range of topics organised in 20 sections that we consider to represent the principal child‐centric and societally relevant themes of microbiology. The topics covered provide a balanced view of positive and negative activities and consequences of microbes, and so should help counteract germophobia. In this regard, the section on microbial well‐being is meant to emphasise that microbes are like humans in that they have health/stress issues. The section on *Microbial Gifts* is rather extensive and reveals the amazing spectrum of biotechnological applications that can and do benefit humankind and the biosphere, some of which can be deployed to help solve or mitigate many of the challenges we and Planet Earth face.

**Table:** 

The TF Sections	
*1. Our Plants* *2. Our Animals* *3. Our Food* *4. Ourselves* *5. Our Well‐being* *6. Our Infections* *7. Our Planet* *8. Global Warming* *9. Our Water* *10. Global Microbiology* *11. Adventures and Discovery*	*12. New Frontiers* *13. Microbial Gifts (Biotechnology)* *14. The Future* *15. The Past* *16. Our Civilisation and Culture* *17. Our Microbial Friends* *18. Microbial Well‐being* *19. How We Study Microbes* *20. Why We Need to be Microbiology Literate*.

#### The structure‐content of a topic framework

**Structure–content of a TF**

- Title page: visual introduction of the topic with an image and question that establishes relevance
- Storyline: summary of microbiological content and its relevance to SDGs, to enable rapid screening and selection of appropriate TFs by educators
- The core of the TF: treatment of the microbiology issues relevant to the topic
- Relevance to sustainability of the microbiology issues covered
- Identification of decision issues at the personal, community and national levels benefitting from a knowledge of the microbiology presented
- Discussion topics and exercises to illustrate stewardship and stakeholder issues impacted by the microbiology
- Teaching aids and supporting materials that complement the microbiology information presented (*some of these will be supplied later, independently of the TF itself*)
- Glossary that explains key terminology.

Key characteristics of TFs are that they are:

- generic. Generic means that much of the information provided in a TF can be adapted by educators to different settings, age groups (including adults at all levels and, in some cases, pre‐school children) and children with different teaching needs. In general, information elements are provided in discrete, bite‐sized pieces, which facilitates the process of selecting some but not necessarily all for any given class. The educators are the TF interpreters and decide which content of a TF will best satisfy the learning goals they define. While not all topics are suitable for the entire span of learner groups, the number and diversity of topics available provides something for everyone.
- essentially stand‐alone. This means that educators do not need to structure knowledge acquisition pathways according to a sequence predetermined by someone else (that said, suggestions will be provided for selections and learning sequences for educators who desire them). As a consequence, educators and learners in different settings can create their own learning paths and timelines and focus on just what is particularly interesting and beneficial for them. Educators teaching in different cultural settings can readily assemble a curriculum specifically tailored to their target audience simply by selecting what they consider to be appropriate and relevant.

The stand‐alone nature of TFs entrains some redundancy, but this also reinforces key messages, which we consider to be an advantage rather than a disadvantage. Further, such redundancy highlights the interconnectedness of TF topics and emphasises the impacts microbes have on different facets of human existence.

- consistent. The style and structure of TFs mostly follow a standard template (but, just as microbiologists cherish microbial diversity, contributed TFs are somewhat diverse in structure, language, complexity and length).
- image‐rich. TFs contain a range of cartoons, photographs and other visual material, both to reveal the invisible subjects under discussion and to illustrate the principal messages.
- relevant to children and society. TFs deal with issues impacted by microbial activities that are relevant, exciting, or fascinating to children and adults. Their goal is to inform about microbiology issues that touch their lives (see Data S1), to provide knowledge and understanding that children should acquire for passage to adulthood, to explain the responsibilities that this engenders and to lay the foundation that will enable them to make informed, best practice decisions.

**Key characteristics of TFs**

- Engaging
- Image‐rich
- Student‐/society‐centric
- Provide context
- Reveal connectivities and interdependencies
- Interdisciplinary and integrative
- Stand‐alone
- Generic
- Consistent
- Stimulate class discussions and interactions

To illustrate the relevance of microbial activities discussed in TFs and their connections with societal issues and decisioning at all levels, we may consider the example of a TF on the subject of Pet Dogs (which is also the template TF). The subject is obviously child‐centric (not microbiology‐centric)–many children would like a pet dog or already have one–and microbiology‐rich. Any family discussion of the pros and cons of acquiring a pet dog will benefit from knowledge of the microbiology. The microbiology content of this particular TF includes:

- pet dog enrichment of microbiome diversity of homes and families and the importance of microbial diversity for the development of immune systems of young children (Mulder et al., 2011; Okada et al., 2010; Rook, 2023)
- infections, some of which are zoonotic (and hence have relevance to COVID‐19: Brüssow, 2023; and One Health: https://www.fao.org/3/cc2289en/cc2289en.pdf)
- infection prevention by vaccines/vaccine‐induced immunity
- climate and environmental footprints of dog food production (Swanson et al., 2013)
- dietary energy and agricultural resource commitments of dog food production (Okin, 2017)
- pollution of public spaces by dog faeces and urine and of underlying aquifers by nitrogen (meat‐rich diets) and phosphorus (‘give a dog a bone’; Hobbie et al., 2017)
- connectivity‐interdependencies: the chemical industry (agrochemicals)–mining industry (phosphorus)–energy industry (supply of the various industries with energy, but also agricultural biogas production)–environmental pollution–biodiversity–food production–health.
- Sustainability aspects discussed that are impacted by the microbiology issues treated are SDG2 (Zero hunger), 3 (Good health), 6 (Clean water), 7 (Affordable energy), 8 (Decent work), 12 (Responsible consumption and production), 13 (Climate action) and 14 (Life below water).

Educators can readily elect to include all these elements in their lesson, or select individual elements, such as microbial diversity and microbiomes, infections, the interface of agriculture and environmental protection, climate change, etc., or combinations thereof. They can also approach the topic from different viewpoints, e.g., food security, One Health, biodiversity, etc. This emphasises the flexibility of TFs, which facilitates their use for different age groups, settings and learning goals.

- interdisciplinary and integrative. Because TFs deal with individual‐/society‐centric issues, they contain and integrate diverse aspects from different disciplines that are relevant and appropriate. For example, an explanation of pathogen transmission in a pandemic–highly topical at the moment–includes a simple mathematical explanation of the Basic Reproduction Number–R_0_–how many new people are infected *on average* by one infected person in a completely susceptible population (a measure of how contagious an infectious disease is) and modelling of infection dynamics, which is important given the central roles of mathematics, statistics, informatics and computation in a vast swathe of current endeavours.
- provide context. In addition to the microbiology presented, TFs place the material in the wider context of societal, regional and global issues. TFs capture recent events to contextualise their content as much as possible (e.g., the COVID‐19 pandemic).
- reveal connectivities and interdependencies. TFs aim to reveal interdependencies of microbial processes, promote systems thinking for everyday considerations, foster critical analysis of relevant parameters in decision‐making and help develop problem‐solving skills.
- include an Evidence Base. Each TF ends with a list of materials for additional reading and exploration, such as short non‐specialist videos/cartoons that explain specific points of learning, general texts and some more scientific reviews and articles for those who would like to explore further. These may cover a range of knowledge and expertise and are meant to be a starting point for educators and learners to further engage in the subject.
- are intended to be available as online video presentations. TFs are supplied as web resources in text form. However, the IMiLI intends to progressively supply them also as online videos for web‐based and offline learning and, alternatively, for television‐based learning for communities lacking stable high‐speed Internet. Provision of teaching resources online will allow continuation of classes during periods of educator absences, enable children to keep up when unable to attend classes and aid educators to prepare lessons.

**FIGURE 1:**
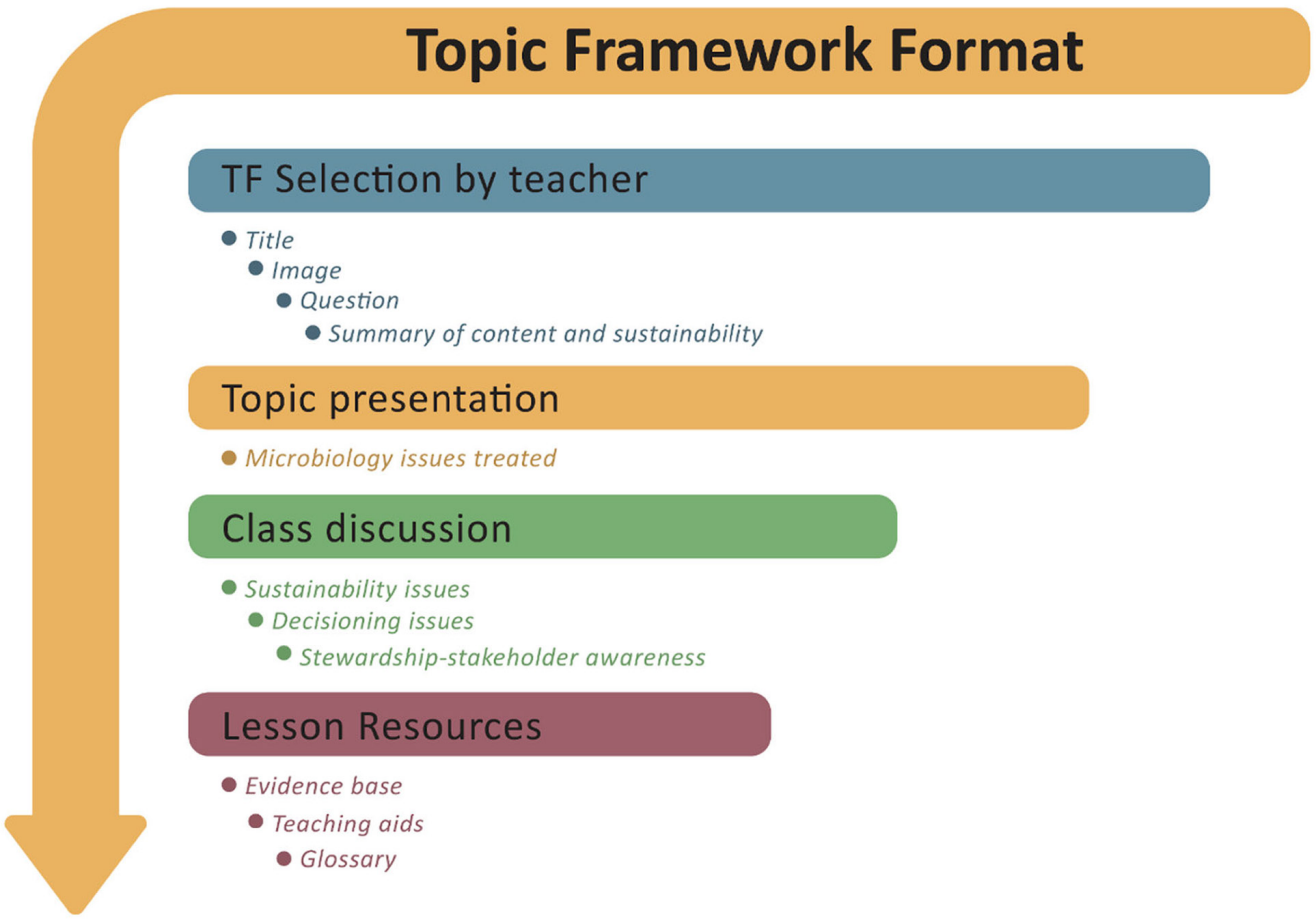
The rationale of use of TFs.

The rationale of the use of TFs is summarised in Figure 1.

### MicroStar portrait galleries

While the focus of the curriculum is on microbial activities that affect us and the wider world, such activities are carried out by specific microbes and microbial communities. Children usually want to know who is responsible for the action. However, unlike a favourite football team consisting of a limited number of players whose names, faces and personalities may be well known, the microbes underlying the activities revealed in the lessons are mostly very numerous and poorly distinguishable. Actors may also be redundant, some working under certain conditions while others rest, with the others taking over under different conditions. Moreover, most microbes have no obvious personalities or recognisable characters. But children love personalities–for example, music and sport stars–to the extent that some are role models. The IMiLI solution is to give due recognition to the *Stars/Heroes/Villains* of the most important processes discussed in the TFs, through portraits that endow them with personalities, some of which are unapologetically anthropocentric or anthropomorphic, for the purposes of illustration and enjoyment, and thus memorability. The Stars have nicknames, such as Alca for *Alcanivorax borkumensis*, because complex Latin names that can change (Spiru, originally for *Spirulina*, which is now *Arthrospira*), should not detract from the fun of discovery. The Galleries of portraits constitute microbial *Halls of Fame*.

The Portraits, mostly about one page of text plus images, are short exposés that allow children to get up close to and learn important details about the actors carrying out the activities described in the TFs, to discover the stars and rogues of the microbial world and become familiar with their characters, characteristics and processes in which they are involved.

The main criterion for inclusion in the Galleries is the strength of the *claim to fame–*the fascination of the story that can be weaved–which is specified up front and creates the drama and whets the appetite for discovery that can excite children and adults alike.

There are a number of different Portrait Galleries focusing on the Stars of different activities/processes, such as biotechnological applications (DesignerMicroStars), infections (MicroRogues), activities that protect us or other organisms from harm (MicroDefenders). In addition, the same format is adopted to profile microbial activities themselves (Fermented foods), microbial products (MicroVaccines) and even vital things microbes need (Elements of life) and the main discoverers of microbes and microbial processes (MicroDiscoverers; MicroNobels).

**Table:** 

Examples of the portrait galleries being created	
**Microbes** *MicroStars* *MicroDefenders* *MicroAgrobiologicals* *DesignerMicroStars* *MicroPests* *MicroBioenergyHeroes* *MicroLabRats* *MicroStars‐That‐Engage‐Our‐Senses* *MicroRogues* *MicroPredators* *MicroWeirdos*	**Microbial topics** *Elements of life* *Earth‐changing MicroActivities* *Humanity‐Changing MicroGifts* *FermentedFoodStars* *GloriousMicrobialMedia* *MicroDiagnostics* *MicroMedicines* *MicroVaccines* *MicroPlasmidStars* *MicroPlasmidStars* *MicroNobels* *MicroDiscoverers*

The template of Gallery portraits is the MicroDefender Alca (*Alcanivorax borkumensis*) whose *claim to fame* is defending the marine biosphere and its resources against ecological damage caused by oil spill pollution. Its activity has wide‐ranging consequences, including defending marine biodiversity and food webs, food security (fishing and aquaculture), employment (in the fishing industry, as well as in tourism) and, importantly for children, defending against beach pollution. Its activities have implications for several SDGs, including *No poverty* (SDG1), *Zero hunger* (SDG2), *Decent work* (SDG8) and *Life below water* (SDG14).

**Some uses of MicroStar gallery portraits**

- Teacher topic preparation
- Student reading: in class, for homework
- Student discussion/presentation: in class
- Student writing: summarising, comparing, assessing, in class, for homework
- Student/class projects: knowledge deepening‐expansion through individual and class web‐based research projects; in‐class presentations of findings as individuals and groups
- Class competitions and debates: between individual students or groups
- TV/movies/social media/music: collecting and writing about/discussing topics featuring involvement of a MicroStar (e.g., Sánchez‐Angulo, 2023)
- Creative arts: depiction of a favourite MicroStar through drawings, paintings, short stories (fact/fiction), videos, comics, theatre, songs, dances, board games
- Home projects involving family and/or friends
- Citizen science international competition

Many science, technology, engineering and mathematics (STEM) professionals have difficulty explaining to family and friends in simple language what they do or what their profession is about, despite a strong desire to communicate this. MicroStars are particularly useful for explaining interesting and relevant aspects of microbiology. For this reason, MicroStar portraits are made available to learned societies for publication in their regular newsletters. The Spanish Society for Microbiology (SEM) publishes MicroStars in Spanish on a monthly schedule, the first of which, Alca, can be viewed as item 10 in the October 2022 SEM Newsletter (https://www.semicrobiologia.org/en/revista‐noticiasem/octubre‐2022).

### Class excursions

A series of recommendations for class excursions has been previously published as an editorial in Microbial Biotechnology (McGenity et al., 2020). These are designed to immerse children in diverse examples of natural and practical microbiology that can be experienced in their localities and explained by educators or local experts, in order that children (and, collaterally, their families and friends) mentally connect everyday observations and experiences with the underlying microbes and their activities. Class excursions may also inspire those who do not always thrive in the traditional classroom. Examples of excursions include wastewater treatment plants, food manufacturing facilities, research centres, geologically impressive landscapes, ponds, heritage works, farms, garden centres, forests, coastlines and even the local market/grocery store (e.g., the cheese counter, freezer: ‘how many microbes did you consume yesterday?’) or pharmacy. There are also recommendations for how to incorporate microbiological elements into visits to museums, zoos and botanical gardens, thereby broadening their scope. As well as focussing on environments local to the school, which makes excursions affordable, opportunities (sometimes involving external professional microbiologists) for bringing remote or exotic environments and microbiological research into the classroom are considered. Excursions can also expose children to career/employment options that may not be on their radar screens and potentially create contacts with professionals who can provide expert information and facilitate exploration of opportunities.

### Multimedia teaching aids

As stated, there is a serious need to visualise, ideally in an interactive fashion, the subjects of lessons. On one hand, there already exists online an enormous selection of MTAs that can be considered for this purpose and, indeed, many TFs include such materials. On the other hand, many existing MTAs are not ideal, either because their purpose is more to promote than to teach or are not of the necessary degree of interest (generated engagement and excitement), quality, clarity, or coherence. It is the intention, therefore, to create custom TF‐specific MTAs. For this purpose, the IMiLI is producing guidelines for the production of different types of MTA: videos, animation, comics, games and so on. A summary of this was published recently (https://www.the‐microbiologist.com/issues/the‐microbiologist‐december‐2021/114.article, pp. 33‐35) and the full analysis will be submitted shortly (van Beek, R., et al., in preparation). The creation of MTAs will generally follow the finalisation of the corresponding TF. Feedback from academics suggests that a number of MTAs will be created by university students, either as coursework or research projects, or as voluntary contributions independent of their studies. It is also anticipated that students in secondary education will create MTAs.

### TF‐specific class experiments

‘Students should have opportunities to participate in authentic research experiences and learn how to evaluate complex biology problems from a variety of perspectives, not just recite facts and terminology.’ Although this quote from Woodin et al. (2010) refers to teaching undergraduate students, it is equally valid for younger and older students. A series of class experiments is in the planning that will specifically address the intention to immerse children in microbiology. When children carry out experiments, they are actors in the learning process, face the reality of it, develop abilities and qualities (planning, structuring, objectivity, observation, analysis, synthesis, teamwork, relationships, communication, etc.) necessary for science, but also life (a blossoming of intelligence, as described in the ‘A comment on education and epistemology’ section of Hallsworth et al., 2023). The themes of class experiments will mostly relate to specific TFs (e.g., bread making/fermentations and preparation of Winogradsky columns) and hence satisfy the need to visualise and personally experience underlying microbial activities discussed in lessons.

**The importance of hands‐on class experiments**

- Physical interaction with the study objects
- Active, personal engagement in the educative process
- Diversity in teaching methods benefiting children with diverse learning needs
- Gain in understanding of the scientific method: identification of the question, design of the experiment to address the question, the importance of controls to validate the results and constrain the range of possible interpretations, carrying out the experiment, discussion and evaluation of results, derivation of conclusions, etc
- Excitement generated during the inception and evolution of the experiment
- Communication of results: preparation of written report, class presentation
- Experiencing teamwork and its value, distribution of responsibilities
- Learning about objectivity and critical analysis of experimental conduct/results by self and others.

### Virtual participation in field work/sampling campaigns in exotic habitats

Microbiology research can be tremendously exciting, especially when it involves field work in fascinating local and/or exotic places, like the polar regions, glaciers, the deep sea, coral reefs, the deep subsurface, hot springs, the Atacama Desert, Rio Tinto, Dallol Volcano, Shark Bay, Cuatro Ciénegas, Pitch Lake, the Amazonian forest and many, many more, which are often expeditions of exploration and discovery (witness the excitement of recent deep sea sampling campaigns involving human‐piloted submersibles, like Alvin (https://www.whoi.edu/what‐we‐do/explore/underwater‐vehicles/hov‐alvin/paged‐2/5/) and remotely operated underwater vehicles, like Jason (https://ndsf.whoi.edu/jason/) and Nereid (https://www.youtube.com/watch?v=rBU8hFz‐4tc).

The spirit and excitement of discovery, and the enormous range of very diverse habitats worked in and sampled, is an exceptional privilege of the microbiology research community that few other disciplines can provide. We have an undeniable responsibility to share this privilege with the general public, not only because they fund such work through the taxes they pay. This responsibility is very well fulfilled by larger organisations like the Woods Hole Institute of Oceanography (link above) and the Fondation Tara Océan (https://fondationtaraocean.org/en/expedition/tara‐oceans/), but less so by smaller organisations and individual research groups.

And we have a special responsibility to communicate this excitement to children, to expose them to the beauty and thrill of things they would ordinarily not see and to satisfy and fuel their thirst for adventure, exploration and discovery in microbiology. This is the reason we have created TF Section 11: *Adventures and Discovery*, which aims to describe a wide range of exotic and fascinating habitats that are explored and sampled by microbiologists and reveal what microbiologists discover with the samples they obtain. And to leverage the content and excitement generated by Section 11, the IMiLI intends to involve children in a more personal, immersive fashion in the most exciting sampling trips through creation of its MicroExplorer series, videos that will transform them into virtual microbiologists on campaigns.

### Home assignments, family–friend and citizen science projects, competitions

As for any other subject, home assignments can be set and suggestions for these will be made, some of which will be centred on the study, use or creation of MicroStar portraits and independent researching of everyday issues on television/social media, etc., having an association with microbes, such as researching/documenting/creating stories around microbes and music (e.g., https://lyrics.lyricfind.com/en‐GB/lyrics/dionne‐warwick‐ill‐never‐fall‐in‐love‐again‐1; https://www.youtube.com/watch?v=oN4Bkal4eFU).

In addition to normal home assignments, the IMiLI will propose facultative family–friend projects for children interested in developing collaborative projects with others. These activities can be carried out as either stand‐alone or networked citizen science projects (research conducted as collaborations between scientists and the wider public), to encourage microbiology‐centric, out‐of‐school activities and to leverage the educational multiplier role of pupils (Timmis et al., 2020).

This type of homework and individual–family–friend projects can be assessed by the educator, presented in class, or be part of competitions: class, school, inter‐school, national, international. In general, children love to work together and also to compete and win prizes (https://en.wikipedia.org/wiki/Gamification), so the motivation for engagement in projects should, where appropriate within the framework of competitions, be increased through awarding of prizes, however modest (e.g., points, levels reached, badges).

An important life lesson that can be learned by being involved in cooperative activities, especially those involving people from very different backgrounds, is that other people often bring new information, ideas, viewpoints and insights to the table that enrich creativity and problem‐solving processes. Moreover, the peer pressure of social media often engenders the pursuit of unattainable perfection to avoid negative reactions and judgements. Working in a group on a common project reveals the ‘normality’ of imperfection among peers, that the goal should not be perfection but rather doing the best possible and that important synergies arising from cooperations often yields a better result than expected from the viewpoint of the individual.

**Table:** 

Aims of the characteristics of the IMiLI teaching resources	
Resources	Purpose/Aim
Free availability for non‐commercial use	Global democratisation of societally relevant microbiology knowledge
A single set of resources for all age groups and teaching goals	Coherence, continuity, ease of transfer among educators, simplicity for life‐long learners: a one‐stop source
Provide knowledge of the workings of the biosphere, our bodies, our origins, our future	Understanding of biology, well‐being, our place in the world
Emphasis on exploration–discovery	Stimulation of excitement, curiosity, motivation
Teaching critical thinking	Informed decisions; countering bias
Teaching systems thinking	Appreciation of interdependence; improving problem‐solving effectiveness
Teaching stewardship and stakeholder participation	Creating awareness; promotion of civic responsibility
Teaching sustainability and relationship to decisions	Improving human, animal, plant, environmental and overall planetary well‐being
Teaching microbial technologies and their deployment to improve social equity	Revealing strategies and mechanisms to level up and reduce asymmetries in access to basic services
Involvement of family–friends	Amplification of interest and involvement; civic engagement
Networking: local–regional–national–global	Amplification of interest and involvement, accessing complementarities/synergies

**FIGURE 2:**
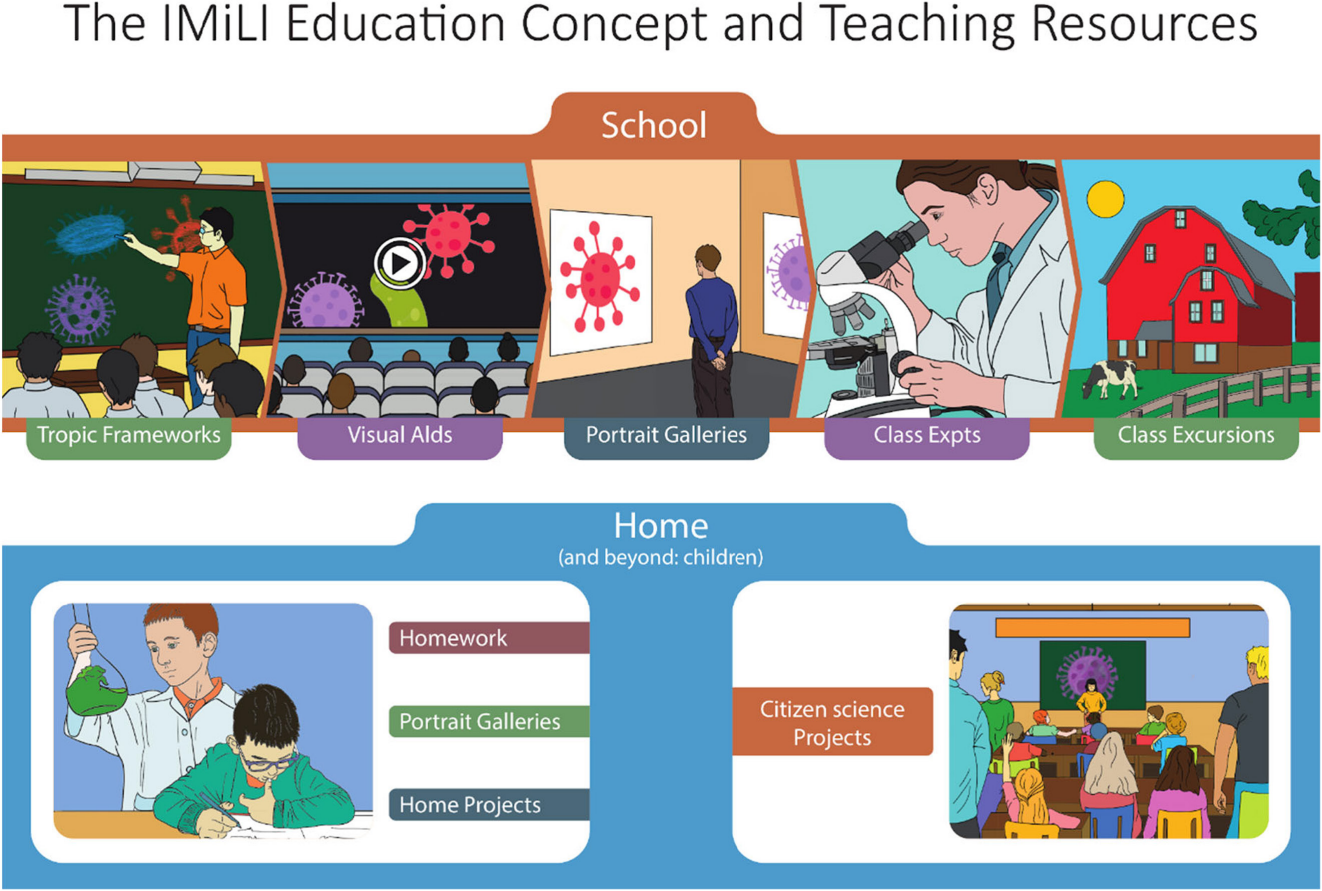
The IMiLI teaching resources and their deployment in microbiology education.

The IMiLI teaching resources and their deployment are summarised in Figure 2.

## REGIONAL CENTRES: THE INTERFACES BETWEEN CREATORS AND USERS OF TEACHING RESOURCES AND NODES OF THE IMiLI ECOSYSTEM

The IMiLI resources are being created in English and need to be translated into whichever language is appropriate for any given setting. Moreover, schools, curricula, educational needs and approaches to teaching vary across the world and the integration of IMiLI resources in diverse educational systems will require local and regional adaption and support. There also needs to be a local interface between the IMiLI teaching resources and end users, education authorities, government, commerce, funding agencies and philanthropic organisations, etc. To provide this, the IMiLI is establishing IMiLI Regional Centres whose responsibilities are summarised below.

**Regional centres**

- adapt IMiLI resources to the regional needs
- translate adapted IMiLI resources into regional languages
- create a professional regional language website posting IMiLI resources
- network with other Regional Centres; sharing translations to serve multi‐cultural regions/nations
- support educators using the IMiLI resources
- develop regional networks of educators to promote the teaching of the IMiLI microbiology curriculum in schools
- facilitate exchanges of experience
- organise workshops, bootcamps that promote and facilitate the use of IMiLI resources
- develop a platform to encourage the exchange of ideas about excursions, experiments and projects
- collect and analyse educator–pupil feedback and communicate to teaching resource creators
- provide feedback on regional needs of IMiLI to maintain the topicality of resources and practises into the future
- organise regional networks of professional microbiologists to promote and facilitate engagement in microbiology outreach activities
- recruit additional teaching materials, where needed or useful
- obtain financial and logistical support for regional IMiLI tasks, where needed
- engage and coordinate with regional and national education agencies and ministries

The IMiLI websites of Regional Centres will develop into dynamic communication/interaction/networking platforms, interfacing with educators, educational agencies and ministries, the wider public and provide an IMiLI ‘info channel’. The Regional Centres will maintain the relevance of IMiLI over time by ensuring that changing needs of teachers, learners and societies are catered for within the evolving IMiLI ecosystem of services and resources. Parts of the websites will be devoted to the direct engagement of self‐learners–children and adults–through the dissemination of microbiology knowledge and news, and the creation of fun microbiological activities and entertainment. They will participate in the processes of recruiting additional teaching materials, including MTAs and games and of editing and harmonisation of IMiLI education resources. They will also collect and analyse teacher–pupil feedback and communicate this to teaching resource creators.

The first Regional Centre, IMiLI‐South Asia Centre (IMiLI‐SAC; imili.org), New Delhi, was created in 2022; the second, IMiLI‐East Asia Headquarters (IMiLI‐EAH; imili‐eah.com), in Suzhou was launched in December 2023. Other Regional Centres are currently under discussion.

## WIDER USE OF THE IMiLI TEACHING RESOURCES

While the IMiLI is focused on early‐life education, the IMiLI teaching resources are generic in design and so can be used for all categories of learners, both children–the next generation of adults–and the current generation of adults. Thus, with some minor tweaks in presentation, they will also serve for adult education/life‐long learning in the classroom and web‐based self‐learning by both adults and children.

### Microbiology in universities and colleges

- Biology for educators in higher education and teacher training courses. The IMiLI resources are ideal for teacher degree courses because they provide prospective educators with the knowledge of microbiology they need, show how to present and convey complex, technical issues in simple attractive ways and provide precisely the teaching resources they will need as educators. Future educators will learn the subject matter with the same materials they will use to teach it.
- Microbiology degree courses. While microbiology courses at college/university are well established and served by excellent texts, like Brock Biology of Microorganisms (Madigan et al., 2021), the individual‐/societal‐centricity, content, spectrum and emphasis of IMiLI resources, such as the TFs, may encourage some college/university teachers to consider modifications to curriculum content, approach (the 3 E's and 3 I's) and emphasis (students are often informed about what they will learn but not always why). Emphasising the importance of interdependencies in processes, with changes often provoking unanticipated compensatory reactions and decisioning, may gain traction, not only in the context of One Health. The IMiLI philosophy of approaching the curriculum from the standpoints of societal relevance and social equity, as well as emphasis on the broader context, the SDGs, relevance for decisions, etc., may give additional impulses to concept development for college/university learning experiences. For example, medical/clinical microbiology courses often lack the concept/context of the metaorganism/holobiont (though there are initiatives aimed at correcting this, e.g., see https://cifar.ca/research‐programs/humans‐the‐microbiome/), the gut‐brain axis and all the other human microbiota‐human organ axes, and human well‐being being dependent upon microbial diversity and the microbial ecosystem, so the IMiLI concept may encourage the incorporation of such aspects.

While information provided in IMiLI resources is written in simple language understandable by educators at a level suitable for transmission to school‐age learners, the messages are universal and thus relevant to higher level education. It is relatively easy to increase level and complexity of IMiLI resources, also to keep up with advances in knowledge, for university‐level teaching (less easy to lower complexity, which is what the IMiLI is doing).

*Microbiology research projects and coursework*: undergraduate and postgraduate students in some universities are already being offered the creation of IMiLI teaching resources as projects and coursework. For example, a trial group of year‐2 undergraduates designed games, posters or quizzes based on a TF of their choice that could be used by teachers to enthuse school children of a defined age. With the exception of mature students, university students were themselves recently school pupils, so are in the best position to judge and create what they would have liked to have learned at school.

- Elective and mandatory courses in the framework of other degree subjects. Since microbes impact a wide range of activities and processes, the teaching of relevant aspects of microbiology is essential in a number of other bachelor's and master's degree courses, including medicine, nursing, veterinary medicine, dental medicine, public health, biological sciences, zoology, botany, biotechnology, environmental sciences, astrobiology, agriculture, bioengineering, energy, geosciences, food science, materials science, business and economics and more. And, as is the case for schoolchildren, it is important that university students not in the STEM fields develop scientific literacy and knowledge to evaluate media coverage and critically analyse and discuss social issues and concerns relevant to the field of microbiology, including disease and pandemics, antibiotic resistance, the ubiquity of microbial products and humankind's exploitation of the microbial world. In most cases, such students will not have a background that facilitates learning formal microbiology and, in some cases, they may have little science knowledge. The availability of teaching resources in language understandable by the general public will facilitate the creation of courses for non‐scientist students.

There is also an increasing tendency to make available non‐specialist courses in climate change/sustainability and related topics to all undergraduate students (e.g., https://www.theguardian.com/world/2022/nov/12/barcelona‐students‐to‐take‐mandatory‐climate‐crisis‐module‐from‐2024; Reimers, 2020; Filho et al., 2021). The IMiLI teaching resources may provide both a model for such courses and a substantial contribution to their content.

### Lifelong learning–training in a global community of scholars and curiosity‐driven information searches

We anticipate that the freely available resources will become the go‐to source of citizen‐relevant microbiology, including the microbiological basis of/impact on pandemics, like COVID‐19 and antimicrobial resistance (Coque et al., 2023); crises, such as global warming (Heleno et al., 2020); and good health, like vaccines, probiotics and feeding our microbiomes (see Data S3), but also of how things work, how we explore the possibility of life on other planets and how we discover new products and processes that can serve humanity.

### Outreach and science communication by academic microbiologists

Many of the contributors of IMiLI teaching resources are already involved in different forms of outreach, such as occasional teaching in schools or in public events such as Open Days of universities and other institutions. These activities are important because they seed interest in microbiology among children, educators and the general public and are therefore pioneering activities. However, adapting academic educational resources to produce new outreach materials involves significant time and effort that many overstretched academics can ill afford. The IMiLI has already invested and continues to invest in this effort to create resources designed for educators and learners. These efforts have created an extensive collection of off‐the‐shelf resources describing the most important and relevant microbial activities (Gilmore, 2021). The IMiLI resources thus remove a significant hurdle to engagement in outreach activities and so should encourage more academics to become involved.

In addition, the IMiLI resources represent an engaging and comprehensive selection of off‐the‐shelf materials for science communicators, thereby expanding the repertoire of topics they can articulate in their efforts to bring societally relevant microbiology to society.

**Wider use of the IMiLI Teaching resources**

- In universities and colleges
  - Teacher training degrees
  - Biology degrees
  - Microbiology degrees (majors and minors)
  - Microbiology degree projects and coursework
  - Elective/mandatory courses for other degrees
  - Elective/mandatory courses independent of degree courses
- Lifelong learning (formal/informal)
- Outreach by professional microbiologists
- Science communication

## KEY ASPECTS OF THE IMiLI CONCEPT MERITING FURTHER DISCUSSIONS

### The 3 R's: relevance! relevance! relevance! Excitement, the fun factor, motivation and learner‐centricity

The goal of the IMiLI curriculum is to transmit information about microbes and their activities that is needed to gain an understanding of ourselves and our place in the biosphere, how things work, what we need to do to maintain and improve our well‐being and that of other inhabitants of the planet and what can go wrong and what needs to be done to correct things when they do. Thus, the curriculum focuses on microbiology topics and concepts *relevant* to the well‐being of individuals, society, other life on the Earth and the planet and its various planetary systems, including climate.

Today, children of many societies spend a considerable amount of time engaged with screen technologies, including electronic games (https://en.wikipedia.org/wiki/Gamification), television and interactive and social media. To engage them and sustain their concentration, the material presented must be relevant to their preoccupations, exciting and tap into their natural fascination for exploration. On the other hand, there is significant global disparity in the life experiences of children in different regions of the world, so the content and design of IMiLI teaching resources seek to captivate and motivate diverse groups.

**Emphasis on relevance in IMiLI teaching resources**

- Learner centricity
- Personal well‐being
- Planetary well‐being
- Understanding processes that affect us, directly/indirectly
- Sustainability
- Exploration, discovery, energising natural curiosity
- Strengthening links between education and communities.

### The notion of *generic*: age dynamics

It may seem obvious that we cannot provide teaching materials that are suited for all ages, from pre‐school to high school! Or can we? Clearly, some topics are too complex for younger children, but others can be taught with different levels of information content, emphases and complexity and so a single knowledge outline/framework could in principle be sufficient for educators to teach multiple age groups. In this regard, it should be noted that, in some parts of the world, especially in remote villages, there may only be one teacher for different classes and age‐groups and all pupils of the school may be taught together in the same classroom. A single, generic teaching resource, adaptable to different age groups, may be particularly advantageous in such situations and may even enable different age groups in the same room to link up occasionally and participate in common activities. It may also encourage older pupils to contribute to the teaching of younger ones.

And is there an abrupt transition from high school to college/university? Is a 15‐year‐old not able to grasp some of the concepts taught at college/university? And, conversely, aren't some topics destined for 15‐year‐olds equally interesting and relevant to college/university students? The IMiLI teaching resources are predicated on the principle that many issues are of generic interest to children and adults, while recognising that not all resources will be suitable for all ages. And the advantages are not inconsiderable.

**Merits of a single set of teaching resources for all learners**

- a single, coherent set of teaching resources that can be used across all educational levels, including that of adults within and outside the framework of universities
- a single, coherent set of resources for educators to grasp and familiarise
- reinforcement of the knowledge and understanding as children progress through school and are provided with more information about the topic from the same resources
- facile evolution and updating of IMiLI resources
- straightforward engagement of educators and pupils in this evolution and hence facilitation of their stakeholder responsibilities and empowerment

### The issue of stand‐alone topics

Formal education in schools, colleges and universities typically follows a determinative sequence of knowledge acquisition, in which understanding of new information necessitates understanding of previously acquired information. The rationale and advantages of this are obvious and it is difficult to imagine an alternative for subjects like languages and those with an inherent chronology, like history, geology, mathematics, etc. However, for other topics, a flexible knowledge network‐based modular approach might be worth considering.

Advantages of a curriculum based on stand‐alone lessons are significant:

- Learner‐adaptable content. The traditional system requires different linked contents for each year. With the type of stand‐alone system the IMiLI is creating, there is a single source of materials for all age groups from which the teacher freely selects content and adapts to whichever class is being taught. This should help motivate educators by allowing them to explore new materials and select just what is most interesting for them, which in turn should generate a more dynamic and inspiring learning environment.
- Culture‐sensitive differences in content. Different cultures emphasise different topics, so different curricula must be developed worldwide. With the stand‐alone, modular system, educators can include cultural criteria in the selection process and so adapt curricula to locally prevailing norms and values and requirements.
- Educator–learner empowerment. The absence of a mandatory sequence of lessons, allowing educators to pick and choose, empowers them. The learners themselves provide the educators with feedback in expressing preferences and critiques, which will undoubtedly influence educator selections, so this also empowers the learners.
- Organic evolution based on educator–learner feedback. Educators are encouraged to provide feedback to the IMiLI and thereby directly influence author revision of the teaching materials and the recruitment of additional TFs on new topics/archiving of less relevant/interesting TFs and hence the dynamic evolution of the curriculum (see also Johnston & Lane, 2021). This represents an important further empowerment of key stakeholders. While educational authorities may wish to maintain certain topics they deem to be essential, teacher–pupil influence on evolution of the curriculum will result in continuous improvement and refining of the curriculum and its thematic relevance.
- Engagement‐motivation. Knowing that feedback will have consequences should motivate educators and learners alike and foster engagement in the topic.
- Advantages for teaching children with different learning abilities or who miss lessons. Some children have difficulties with structured curricula, in which each lesson builds on previous ones, or where the broader relevance and interdependencies of diverse issues are not always clear. Institution of stand‐alone lessons may reduce pressure/stress caused by structured curricula or because children miss lessons, change school and other factors, since missing some lessons does not prejudice comprehension of other (subsequent) lessons. Moreover, since the teaching resources are web‐based and freely available, children have the opportunity to explore and catch up on missed content (see also unesco.org/en/legal‐affairs/recommendation‐open‐educational‐resources.oer).
- Changes in educators or teaching priorities. Stand‐alone lessons facilitate changes in both teaching priorities and teaching personnel because it is not essential that a new educator continues a specific learning pathway which had been adopted by a predecessor.

### Examinations and mandated curricula

While stand‐alone teaching resources and selection of learning pathways have considerable merit, they may be perceived as incompatible with student assessment and examination practices, or strict locally mandated curricula. This issue is particularly relevant where regional or national examinations relate to specific learning requirements. However, when this arises for classes, it can be handled in various ways:

- through designation of some core, examinable topics compulsory for all students,
- by focusing on abilities, through formulating generic/general questions that can be answered whichever topics have been studied,
- through project work, such as selecting a MicroStar relevant to their environment, preparing a report on it, presenting orally in class and
- through a combination of the above.

### The interdependence of things and systems thinking

As John Muir observed: ‘When we try to pick out anything by itself, we find it hitched to everything else in the Universe.’ (https://vault.sierraclub.org/john_muir_exhibit/writings/misquotes.aspx; see also: ‘Restructure curricula to emphasize interdisciplinary projects and connections between disciplines’: https://www3.weforum.org/docs/WEF_Innovative_Learning_Solutions_to_Navigate_Complexity_2023.pdf).

Many leaders confronted with crises tend to view an individual symptom in isolation and develop a response designed to specifically address it, rather than its root causes; they often ignore the fact that, in many instances, a problem faced is inextricably intertwined and integrated with and interdependent on, others. As a result of this *linear thinking*, an implemented solution strategy may fail because of unseen constraints imposed by other factors and may even engender new, unanticipated, adverse outcomes (as Albert Einstein noted: ‘We cannot solve our problems with the same thinking we used when we created them.’). Finding effective solutions to many problems requires *systems thinking*: careful analysis of the system in which the symptom to be addressed is embedded and exploration of relevant networks, including those elements, the influence of which may not be immediately apparent (https://systemthinking.docs.cern.ch; https://thesystemsthinker.com/making‐the‐jump‐to‐systems‐thinking/; https://www.youtube.com/watch?v=FW6MXqzeg7M&t=10s).

Leaders frequently consult experts to inform themselves of the key issues underlying problems. However, without awareness of interdependencies, leaders risk creating a restricted, potentially misleading solution strategy framework for the experts they consult, which may engender decisions based primarily on siloed considerations. Modellers are often consulted to provide predictions of future trends, but their models are only as good as the quality and relevance of the boundaries and information upon which they are based. They also do not always incorporate relevant connected variables. As George Box famously stated: ‘all models are wrong, some are useful’.

To illustrate and emphasise the pervasive interdependence of microbial activities and their influence on problems of current concern, it can be instructive to consider just one topic that is vital to us all, is experienced personally every day and is one of the high priorities of children, namely food (its production, processing, transport, supply to consumers, preparation, consumption, spoilage and fate; see Data S3). This example reveals the web of microbial connections and interdependencies relating inter alia to soil fertility, plant health and resilience to stresses, food‐borne infections, food deterioration, food preservation and upgrading, environmental pollution, eutrophication/oxygen minimum zones/loss of biodiversity, antimicrobial resistance, global warming and energy security.

The issue of interdependence may not always be easy to convey and, more importantly, to instil in the deliberations of children (and adults, for that matter). Nevertheless, it is imperative that those being taught are made aware of it so that at least some will attempt to consider the whole picture when faced with the need for a decision. The curriculum in general and the TFs in particular, attempt, where possible, to emphasise the pervasiveness of interdependence and several TFs explicitly deal with the issue, such as *Global connectivity* and *Interconnected nature of environmental problems*. One, on the microbiology of romance and reproduction, is designed to expose connectivity in a topic that is likely to be of particular interest to most young adults attaining sexual maturity (see also Timmis, 2023). And there will be one or more Gallery Portraits specifically dedicated to illustrations of systems and linear thinking.

### Critical thinking

Knowledge and understanding of issues–in our case, microbiology‐influenced issues–underlying problems faced are the enablers of obtaining and evaluating the specific evidence needed for evidence‐based decision‐making. But they are not sufficient for reaching best option decisions, inter alia because of bias. Bias accumulates with age and exposure to biases of others and is one reason why leaders fail to take prompt effective responses to problems and why the public fails to hold them to account.

While it is not possible to eliminate personal or cultural biases, it is possible to lessen and counteract them by making children (and adults) aware of different types of biases and teaching them evidence‐based deliberation and its value and about misinformation and persuasion by others, so that they as decision takers are more likely to be less biased and stakeholders are more likely to recognise bias in their leaders and hold them to account.

Importantly, optimal decision‐making and problem‐solving (https://en.wikipedia.org/wiki/Problem_solving) are favoured by critical and systems thinking. Critical thinking is the analysis of available facts, evidence, observations and arguments, in order to form a judgement by the application of rational, sceptical and unbiased analyses and evaluation (e.g., see Glaser, 1941; Danchin, 2023; https://www.skillsyouneed.com/learn/critical‐thinking.html; https://plato.stanford.edu/entries/critical‐thinking/).

However, critical thinking is not easy to teach and even more difficult to implement, because it requires both adoption of principles and volition: a certain mental attitude (https://www.criticalthinking.org/pages/defining‐critical‐thinking/766). Especially for younger members of society, it is rather abstract. But, since biases accumulate with age, it is vital to begin as early as possible (Timmis, et al., How to foster critical thinking in school: the microbiologists' approach, in preparation). To confront the conundrum that critical thinking is best taught to older learners, but increasingly compromised by age‐accumulating biases, the IMiLI will initiate awareness of critical thinking in young people by introducing in story form (https://www.forbes.com/sites/shanesnow/2023/01/16/science‐shows‐humans‐have‐massive‐capacity‐for‐sustained‐attention‐and‐storytelling‐unlocks‐it/)–the MicroChats–readily understood practical elements of critical thinking in the context of familiar examples.

**MicroChats aiming to convey the elements of critical thinking: a selection of those in preparation**

- What is the evidence?
- Plausibility and reasonable doubt (if it seems too good to be true, it probably is)
- Correlation and causality
- Bias and misinformation
- Interdependencies and systems thinking
- Limiting parameters/bottlenecks/pinch points: focusing on the key issues
- Essentiality: if it ain't broke, don't fix it
- Want is not the same as need
- Is a decision issue black and white or shades of grey?
- Stakeholder involvement: two(+) brains and diverse perspectives are (usually) better than one
- Due diligence and cost:benefit analysis
- Cost externalisation: a reality check of true costs
- Success: has it been defined and can it be measured?
- Who benefits (directly; indirectly)?
- *Talking the talk* and *walking the walk*
- Experience versus talent
- Benchmarking and best practice
- Transparency and accountability
- Quality control

Stories exemplifying elements of critical thinking also include examples of *uncritical thinking* to reveal the consequences of the latter. (Simply getting into the habit of posing the question *why?* to proposed actions and thereby demanding justification (which may lead to some level of cost:benefit discussion), already transfers decisioning into a more healthy framework.) In this way, children learn to apply critical thinking without the baggage of the theory. Collectively, the MicroChats will also aim to integrate critical thinking with systems thinking and problem‐solving.

**Key role of microbiology education in equipping children with generic knowledge and problem‐solving abilities they will need as adults**

- providing understanding of fundamental issues central to planetary and personal well‐being, including the vital importance of microbes
- revealing the interconnectedness of things and dangers of consideration of topics in isolation (*silo thinking*)
- fostering critical thinking that will aid them strive for evidence‐based, rational decisions and policies
- widening vistas by revealing and stimulating interest in other systems and cultures and the options they offer and stimulating *out‐of‐the‐box* considerations
- encouraging a sense of personal responsibility and involvement for the state‐of‐affairs and its improvement
- inspiring the creativity (artistic and scientific: www.weforum.org/agenda/2023/07/creativity‐science‐matters‐ways‐to‐achieve‐it/), curiosity and initiative that will be needed to address future societal and environmental problems.

### The 3 D's: decisioning, decisioning, decisioning (and the vital importance of sustainability considerations)

Since the primary goal of education is to develop the minds of children in preparation for adulthood and its everyday events and regular challenges and opportunities that require decisions, an emphasis on informed decisioning best practice is essential. Therefore, key to our deliberations on what should be taught is related to decisioning and the critical and systems thinking underlying it. It is also important to communicate the fact that science and evidence are only the best available at the present time and that future developments may provide new evidence that will require decision/policy modification. In any case, the best decisions can also be wrong, so it is important to communicate the need to monitor the efficacy of decisions–*oversight*–and re‐evaluate when needed.

**The 3 D's: Decisioning! Decisioning! Decisioning (see also Data S4: Terry McGenity)!**

- *informing decisioning* (acquiring relevant knowledge and understanding)
- the *act of decisioning* (objectively considering the relevant issues and options to reach a decision and
- *accountability in decisioning* (explaining/justifying decisions and, crucially, taking responsibility for the consequences).

One of the key overarching considerations in policy development is sustainability, which is itself a series of highly interdependent aspects of human behaviour and endeavour. This is readily seen in the sustainability development goals (SDGs) formulated by the United Nations (https://sdgs.un.org/2030agenda) which, though discrete, are obviously overlapping and interdependent (e.g., poverty, hunger, health, employment). While an old and proven mantra is ‘if it ain’t broke, don’t fix it’, when something does look as though it will break, the mantra has become ‘if it needs fixing, make sure the fix is consistent with sustainable development’. It is also worth mentioning: considering sustainability aspects of a decision also helps to counter silo thinking.

### Immersion in the world of microbes, and microbes as teachers

To sustainably engage and motivate all pupils, especially those on the margins–to be maximally inclusive–it is essential to make education fun, fascinating from the point of view of the students, relevant to their lives and experiences and that engages and stimulates their inherent thirst for discovery and exploration. Despite the mostly lack of visibility of microbes, microbiology has huge pedagogical advantages. In teaching microbiology, it is essential to leverage these advantages, in particular the opportunities for immersion and immersive experiences.

**Immersive strategies**

- *Let's make it personal!* TF Sections 4, 6, etc.
- *Let's make it physical!* Class and field experiments
- *Let's make it exciting!* Virtual participation in those incredible research expeditions to/sampling campaigns in exotic places
- *Let's make it interactive!* Class discussions/competitions relating to TF topics, Gallery portraits
- *Let's make it empowering!* Feedback to teaching materials creators; learner creation of new resources
- *Let's make it fun!* Class excursions; microbial games–art–theatre–music
- *Let's make it social!* Family–friend–citizen science projects.

And immersion in microbiology reveals that microbes themselves have much to teach us.

**Microbes as teachers–what we can learn from them**

- Microbes vitalise! They give added value to their habitats (soil, water, air, rocks)
- Microbes function almost entirely in partnerships: partnerships bring together key skills to achieve best solutions!
- Microbes operate at low energy levels (we can certainly lower our energy consumption)
- Microbes integrate processes (and so can we, e.g., integrate CO_2_‐producing processes with CO_2_ capturing/converting processes)
- Circular economy of resources
- Microbial commons.

### Opening vistas for the future

Though not explicitly specified in the goals of education discussed at the outset, education should also open vistas for the future personal development of the learner, from the points of view of both career routes and non‐career activities. The pervasiveness of microbial activities that impact human endeavours and human–biosphere–planetary well‐being has as a consequence that microbiology‐microbial technology knowledge and expertise find application in many different types of careers.

Children may be advised on career options by experts, but most experts have little knowledge of the range of careers that benefit from a microbiology knowledge. To rectify this lack of information, the IMiLI will create a comprehensive series of career portraits–the MicroCareers Gallery–that will profile the diverse microbiology‐relevant careers both for children considering career options and for career advisors. This Gallery will also expose the value of microbiology knowledge for careers such as finance (e.g., venture capital investment in start‐ups) not traditionally considered to require expertise in microbiology, to provide new vistas for children that help them imagine their futures in engaging new ways. If we can stimulate excitement that leads to children deciding to get involved in vector control in Africa, exploration of the deep ocean, creation of new pre‐ and probiotics to treat/prevent mental illness, seek microbes that can degrade and recycle recalcitrant wastes, formulate and implement policies to reduce our negative impact on the planet, etc., we will achieve our goals.

**Table:** 

Some of the diversity of careers exploiting microbiology knowledge and expertise		
teaching and research healthcare (human, veterinary, dental) pharma forensics chemistry personal care products	food industry agriculture environmental protection waste management recycling materials quality control	energy mining construction innovation finance and investment diverse government and non‐government agencies

Providing learners with information and materials that set them on the path to exploration and excitement in discovery, encourages and supports long‐term satisfying constructive and productive endeavours–professional–leisure–family–community–that benefit them and society.

### Engagement, fun, play, creative arts and competitions

Children who are excited by a subject feel engaged. But there are other ways and means of engaging them. Children are worried about the future of the world they will inherit, witness the international Friday school strikes to raise awareness about climate change and stimulate mitigating activities (https://en.wikipedia.org/wiki/School_Strike_for_Climate). They are also interested in sustainability and other societally relevant topics. Microbial activities impact many of these topics, so by dealing with them, the curriculum will engage.

Children are children, each growing their own lived experience and enjoy playing and social activities, being unserious, challenging one another and having fun, so it is imperative that, where possible, education is fun. The diversity and pervasiveness of microbial activities and impacts upon humans, our pets and plants, the planet, constitutes a huge space for the development of ideas for play, artistic creativity and competitions, such as board games, stories and storytelling, theatre, music, petri dish art (e.g., https://twitter.com/hashtag/MicrobeArt2023?src=hashtag_click) and sand art (https://drive.google.com/file/d/1tssshADADckp8HX3ge2FjLZQcb‐FI98Y/view). The IMiLI aims to support educators in this endeavour by providing suggestions for group activities, class competitions, ideas for creative arts, games and so forth. This will be particularly important for the creatives who go cold when they see a science lab (see Data S4: Rachel Armstrong).

And, as also can be seen from the Strike for Climate movement, children enjoy interacting with others, especially via social media and participating in international activities, interactions that can amplify their enthusiasm and motivation. To engage and stimulate this enthusiasm for group activities, the IMiLI will encourage development by IMiLI Regional Centres of family–friend network projects, class and school projects and international citizen science projects and competitions (e.g., https://www.societyforscience.org/research‐at‐home/citizen‐science/; McGenity et al., 2020; Shah & Martinez, 2016; https://tinyearth.wisc.edu; https://igem.org). Such collaborative and competitive activities can significantly amplify the fun factor, social interactions, engagement and motivation of learners and enrich the teaching experiences of educators (e.g., https://en.wikipedia.org/wiki/Gamification_of_learning; https://academictech.uchicago.edu/2021/11/23/introduction‐to‐the‐use‐of‐gamification‐in‐higher‐education‐part‐1/; https://uwaterloo.ca/centre‐for‐teaching‐excellence/catalogs/tip‐sheets/gamification‐and‐game‐based‐learning).

The fun factor can also be elevated by the explicit interjection of humour into teaching resources themselves, where appropriate and indeed some of the IMiLI materials are explicitly humorous (e.g., video on a do‐it‐yourself microscope and some of the *MicroChats*).

Another aspect of engagement (and empowerment) of children is their ability to act themselves as educators (Timmis et al., 2020), as agents of knowledge transmission among family members and friend and peer networks. The pupil/student‐centricity of the curriculum, the portrait galleries that expose fascinating personalities of microbes and the family projects being designed, will all stimulate the natural tendency of children to transmit their newly acquired microbiology knowledge and hence their feeling of empowerment.

### Engendering classroom positivity

A great deal has been written about classroom positivity (e.g., ‘Positivity is the foundation for optimism, which can manifest as reduced stress, greater resilience, better problem‐solving skills and higher levels of achievement. Positive, optimistic individuals often have greater motivation to work through challenges, both in the classroom and in life.’ https://www.pbisrewards.com/blog/build‐a‐positive‐classroom‐environment/) and how it can/should be achieved. However, commonalities include valuing cultural diversity and promoting a feeling of community, encouraging active participation, teacher and mutual support, extending classroom activities outside of school and reinforcing positivity through rewards and incentives for success. While it is not the intention to deal here with the generic issue of classroom positivity, it is important to emphasise how microbiology inherently lends itself to efforts to achieve classroom positivity and how the IMiLI concept leverages this. For example

- the value of diversity: the enormous extent of microbial diversity and its value to biosphere health (and, also the contrary: e.g., the problems of monocultures in agriculture; reducing diversity of microbiomes) is a recurring theme in the IMiLI resources, so it is a natural step to initiate class discussions on this topic and then move to human diversity and its value (how do we differ? Role playing imagining we are like someone else: what new things can we contribute, etc.)
- community: collaborative class/group projects – examples are provided in the teaching resources, including fun class experiments like preparation of dough for bread and pizza and group work in the creative arts, such as theatre, song and dance and in competitions, etc. designed around microbes
- active participation: class discussions/debates on issues suggested inter alia in the TFs, class and home projects including artistic and recreational activities designed around microbes; etc.
- extending classroom activities outside of school: joint projects that also involve family and friends; citizen science projects; class excursions that have been suggested by the IMiLI; etc,

An important aspect of the IMiLI that relates to disadvantaged children and classroom positivity is language. Quite apart from multicultural societies whose schools may handle well (or, in some cases, less well) the teaching of children (and in some cases adults) whose mother tongues may differ from the official national/regional language, current mass migrations are resulting in children being incorporated in significant numbers from one day to the next into classrooms in which lessons are held in a–for them–hardly‐understood foreign language. While children in general quickly acquire new languages, the transition period can be difficult, stressful and may result in some falling behind. Having the teaching materials available in their own languages may reduce this. The different Regional Centres will be creating translations of IMiLI resources which they will post on their websites for anyone to access. So, for example, a Chinese pupil transferred into a German school will be able to access Chinese translations of IMiLI lessons.

And, of course, although classroom positivity is an important issue, some children, such as those in conflict zones, or who are politically constrained, may not have the benefit of a teacher in a classroom. In such cases, where Internet is available, the online availability of IMiLI teaching materials may, for some, enable self‐education and provide opportunities to engage with other like‐minded peers and educators across the globe.

### The biodiversity mindset–appreciation and the respect of human diversity and dignity–and the inclusivity contract

Much tension and conflict in the world is caused by differences (real, perceived or imaginary) that may distinguish one type of person from another, including those relating to biases of personal preferences, gender, medical/genetic issues and disabilities, etc. Some of our commonalities may be minimised and differences maximised, distorted and even weaponised by those who seek to gain and maintain influence and power through divisiveness, demonisation of others and propagation of discrimination (Afshar, 2013; Ejiofor, 2023; Feldman, 2023). A powerful means of counteracting this are efforts to create knowledge, understanding and familiarity of others, reveal and emphasise our commonalities (including our microbiomes), encourage appreciation and respect of the value of diversity and humanity, have empathy for the disadvantaged and promote contact, dialogue and friendship between diverse groups and peoples (e.g., see SDG 10).

Exceptions notwithstanding, biologists in general and microbiologists in particular love and treasure biodiversity for a host of reasons including the beauty of variation in form or function (e.g., flowers, butterflies, sulfur‐oxidising bacterIa), the high stability and functionality of biodiverse ecosystems and the efficiency of resource utilisation in diverse communities (e.g., see Timmis et al., 2023). Microbes inform us of the advantages of living together and of engaging in beneficial transactions with different organisms. *Microbiologists have a special and compelling duty to inform human society of the benefits of diversity and the tragedy of ‘othering’*.

A characteristic feature of the teaching resources provided by the IMiLI is that their authors are clearly specified: educators and learners can see that a resource has been created by a particular expert or group of experts in India, China, the United States, Costa Rica, Mexico, Italy, Nigeria, Australia, Russia, France, Germany and so on, i.e., by people from all over the world and diverse cultures. Since many TF authors are willing to act as mentors and interact with educators and children, at least in the context of the teaching resources they have created and, in some cases, in the wider context of microbiology, there exists the possibility of a teacher/child from Ethiopia being mentored by an Australian microbiologist, a teacher/child from Denmark being mentored by a Kenyan microbiologist and so on. Such interactions will promote the evolution of international networks of microbiology education and citizen knowledge acquisition and exchange and, importantly, create familiarity with other peoples and cultures. These will be reinforced by IMiLI Citizen Science projects, which will not only create common projects in different parts of the world but also provide global social networking opportunities for the children and educators who participate in them. Such elements of the IMiLI concept will, in some cases, result in the blossoming of international friendships and formation of groups with similar interests.

Furthermore, as revealed in IMiLI teaching resources, many microbial technologies, current and in development, e.g., malaria vaccines, frugal diagnostics, seed inoculants, are specifically targeted for regions different from those where they are developed and their deployment involves a diverse spectrum of actors from different fields. And the research and development underpinning such advances is an international endeavour and relies on the input, expertise and resources (natural, intellectual, experience, knowledge) of scientists from different countries and backgrounds representing a diversity of opinions, traditions, cultures, etc. Just as there are interdependencies of different microbes in environmental processes, so there are interdependencies of humans with different skills from different regions and cultures crucial to the implementation of microbial solutions to pressing local, regional and global problems.

The IMiLI teaching resources and activities will thus enable educators and learners to appreciate the nature of science and the importance of diversity – microbial and human – and should help erode perceived differences between peoples and lead to better appreciation of our shared commonalities. This, in turn should help counteract bias, prejudice and attempts to emphasise and distort differences. We hope that the strategic aim of the IMiLI to help fulfil *the inclusivity contract of society,* will eventually be reinforced by the creation of international study programmes/stipends to promote the internationalisation of microbiology education and its democratisation.

### Provision of basic goods and resources, levelling up and social equity

A consequence of integrating the IMiLI resources into school curricula is that children worldwide will learn about the huge diversity of biotechnological applications (and other potential applications) of microbial activities and their ability to satisfy many basic needs, including the provision of clean water, sanitation, vaccines, medicines, food security, new enterprises and jobs and thereby contribute to the provision of basic goods and services considered to be a human right: a *birth right* (Anand et al., 2023). Appreciation of relevant microbiology and social responsibilities gained in school will inform decisions in adulthood to implement measures inter alia to level up, to fight poverty and hunger, to use effective biotechnologies to promote a wide range of improvements in human well‐being and thus enhance human dignity. As we have discussed elsewhere (Anand et al., 2023), asymmetries in basic goods and services can become a source of conflicts, so more fairly (re‐)distributing basic goods and services across the globe can also reduce such conflicts and promote peace and prosperity.

**Potential humanitarian reach of a curriculum in societally relevant microbiology**

Microbes and their activities affect all living beings and thereby constitute the functional glue of the biosphere. Microbes also glue the biosphere to the geosphere and atmosphere. Might the IMiLI, in its effort to reveal the microbial world and how it affects us all, and to microbially engage children (and adults) globally, become an international humanitarian glue? And in emphasising the potential of microbial technologies to provide basic services and goods where they are lacking, and the importance of deploying them, the IMiLI may contribute in some measure to improvement of the human condition and increasing human dignity. While it may be going too far to suggest that the IMiLI will directly contribute to efforts to reduce conflicts in the world, in opening children's minds to the richness and value of human diversity, our similarities and our ability to leverage microbial activities to diminish social inequalities, it should reinforce other endeavours and may well have a cumulative positive impact over time.

### Teaching in informal settlements and refugee camps

The world population is currently around 8 billion. Of these, I billion live in informal settlements (https://gsgii.org/wp‐content/uploads/2022/05/informal‐settlements‐report‐2022.pdf) with another 6.6 million living in refugee camps. (https://www.unrefugees.org/news/refugee‐camps‐explained/). More than 100 million people are classified as displaced, i.e., have lost their homes, and the rising trajectory of human displacement is alarming (https://www.unhcr.org/about‐unhcr/who‐we‐are/figures‐glance). In all these situations, there are large numbers of children whose education is challenging but crucial. According to UNESCO, about 244 million children do not attend school (244 M children won't start the new school year|Global Education Monitoring Report (unesco.org).

While the IMiLI cannot contribute directly to increasing the number of children in education, by creating teaching materials that emphasise child interest and that are relevant to problems encountered in informal settlements and refugee camps, such as water, hygiene, infections, pollution, crops and food, it can stimulate educators and learners, encourage regional would‐be educators to engage, and perhaps stimulate improvements in settlement practices and organisation (Hallsworth et al., in preparation).

### The microbiology education value chain and cloud

The concept of value chains developed by Porter (Porter, 1985; see also https://www.cisl.cam.ac.uk/education/graduate‐study/pgcerts/value‐chain‐defs), deals with the chain of activities that an organisation performs to create a product of value for a customer and the manner in which value is added along the chain. The goal is to identify activities in the chain where optimisation can add value to the system. Value can be financial, but also ethical, environmental, and societal. Customer feedback is often a key driver of value addition.

Education has vital consequences for individuals and society which are sometimes depicted as an educational value chain (e.g., Dorri et al., 2012; Mpofu & Chikati, 2013). In education (and other contexts), value chains, like food chains, are not linear but branched and constitute a web or network, which increases the scope for adding value. Education value chains available on the Internet are often restricted to the educational context–university, school, business. One notion of the educational value chain is: Information–Knowledge–Wisdom–Changed mindset–Changed behaviour–Influencing others to change their mindset and behaviour (https://www.linkedin.com/pulse/knowledge‐wisdom‐education‐value‐chain‐formula‐d/), which has the virtue of emphasising the progression of knowledge acquisition to mindset change, i.e., of being transformative and supporting development of problem‐solving skills.

**FIGURE 3:**
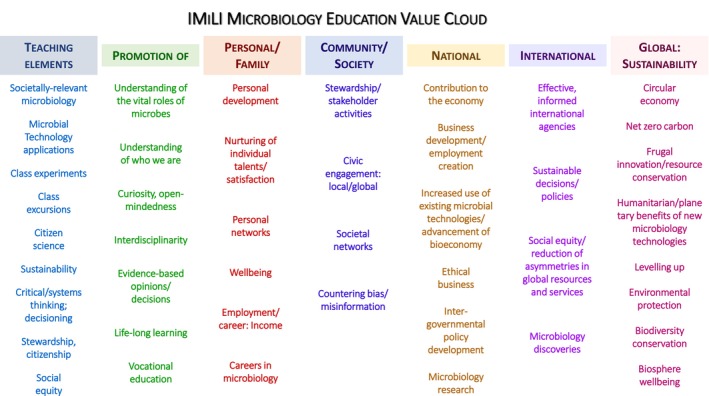
The societally relevant microbiology education value cloud.

But education has almost limitless consequences for humanity and a far‐ranging value web. Figure 3 integrates the issues discussed above into an *education value cloud*. Some of the elements are microbiology education‐specific, but the majority are or should be generic. We elected to depict the microbiology education value cloud in terms of increasing societal scale and complexity, ranging from the values added at the personal level, to those at the family–community–national and global levels, but there are obviously other ways of representing opportunities to add value to education, including a more traditional way involving a branching path.

Intervention opportunities to increase value from education in societally relevant microbiology that are apparent from Figure 3 are numerous. They include increasing emphasis on microbial technology applications to improve social equity, frugal innovation and levelling up, increasing emphasis on critical and systems thinking to reduce bias and the impact of misinformation and to increase the efficacy of political decisions at all levels. As is the case for classical commercial value chains, customer–in our case the educator‐learner–feedback is essential to maximise options to add value.

Another important utility of the educational value web is that it reveals the comprehensive set of values that education embodies. The knock‐on effects of education are all pervasive, ranging from personal development and betterment to consequences for the family and friends, career, the economy, sustainability, societal well‐being and the future of the biosphere and planet. However, despite the obvious societal importance of education, in many societies it is undervalued, as reflected in the facts that teachers are relatively underpaid (compared with say bankers, lawyers) and overworked because of understaffing, their societal status does not reflect the importance of their work and there is serious underinvestment in education infrastructure. Underinvestment in education seriously constrains the development and realisation of inherent potential of young people and, as a consequence, prejudices overall economic performance and sustainability. It is worth noting that subsidies for strategic industries and recruitment of individuals with specific skill sets cannot recoup this lost potential. Given the pervasive power of microbial technologies to solve problems and create novel products and processes, the IMiLI will seek to explore, extend and make apparent to society the educational value chain/web, with the explicit aim of raising appreciation of education and educators and the need to support them better and allocate them their rightful place in society.

## CONCLUDING REMARKS

### The need for change: IMiLI an enabler of change

*Formal education*. Calls for change in school curricula, to make future generations of adults fitter for the challenges they will face, are increasing in frequency (https://widgets.weforum.org/nve‐2015/chapter1.html; https://www.un.org/sites/un2.un.org/files/report_on_the_2022_transforming_education_summit.pdf).). While the case for change is irrefutable, key questions are (i) what new elements (subjects/topics; teaching methods, etc.) are needed, (ii) to what purpose and (iii) how should they be integrated into existing curricula? Much discussion about what changes are needed in education focuses on employment skills needed for national economic development (The reskilling revolution: https://www.weforum.org/agenda/2023/09/universities‐adapt‐future‐of‐learning‐skills‐work/). However, the well‐being of individuals, nations and the biosphere is determined by more than employment and national economies, not least because we and future generations face/will face crises that seriously impact diverse aspects of humanity and the planet. Therefore, change in school curricula is needed not only to improve employability potential, but also to elevate awareness of broader societal and planetary challenges, and understanding of their causes and potential solutions and prepare people for significant behavioural and societal change. And we should not neglect the importance of philosophy in personal development (O'Malley, 2016; O'Malley & Parke, 2020); indeed the philosophy of microbiology is a theme in the IMiLI curriculum.

Many of the problems faced by humanity have microbiological solutions or mitigation actions, problems and solutions that also impact employment and economies. Indeed, the core of the bioeconomy (www.fao.org/interactive/bioeconomy/en/) and much of the Green Deal (www.consilium.europa.eu/en/policies/green‐deal/) is based on microbial technologies. Educating children in societally relevant microbiology will provide them with important knowledge and skills that are key to informed decisions relating to their personal well‐being, citizenship and economic development.

Major societal challenges are worsening inequalities, inadequacies in basic goods and services in impoverished communities, bias‐driven polarisation of opinions and societal fragmentation and disinformation amplified by social media. Such challenges need to be countered by promoting at an early age critical‐systems thinking, global citizenship and the value of diversity and sustainability. While classical teaching also emphasises ramifications and applications of the subjects taught, the resources being created by the IMiLI aim to go beyond this and teach and raise awareness of societally relevant skills and qualities, such as critical thinking and objective, evidence‐based discussion, opinion‐building and decisioning, the interconnectedness of things, systems thinking and problem‐solving, sustainability, stewardship and stakeholder responsibility and activities, global citizenship and responsibilities and the educational value chain.

Current education at all levels is mainly organised along disciplinary lines with disciplinary boundaries often acting as barriers to exploration of transdisciplinary networks. But, life is a web of interconnected experiences, issues to understand and problems to solve, just like that of the larger biosphere of which we are a part. For children to become adults who look beyond the saucer, make connections that are vital to identifying the underlying problem rather than just seeing the symptoms, it is essential that interdependencies are not only revealed to them but shown to be pervasive. This can be done by both dedicated teaching of interdisciplinarity, illustrated by practical, everyday examples and through exposing the connections between disciplines. By dealing with microbial activities that impact societally relevant issues and their ramifications, the IMiLI seeks to counter teaching, thinking, opinion formation and decisioning from disciplinary silos.

*Lifelong learning*. Lifelong learning has always been the activity of scholars and the curious, but is growing in importance for a number of reasons that include opportunity (increasing longevity/longer retirement can provide time previously lacking during periods of employment), greater availability of educational resources (especially on the Internet) and the need to learn new skills for employment (https://www.uil.unesco.org/en/unesco‐institute/mandate/lifelong‐learning). Lifelong learning is also a growing priority of various organisations, including the United Nations, which has included it in its SDGs (*Goal 4. Ensure inclusive and equitable quality education and promote lifelong learning opportunities for all*; ….) and the European Commission (‘To succeed, lifelong learning for all must become a reality in Europe.’ (p. 4: https://ec.europa.eu/migrant‐integration/sites/default/files/2020‐07/SkillsAgenda.pdf). Microbial activities and their manifold impacts on human lives, endeavours and well‐being and on biosphere and planetary health, predestine them to be a priority topic of public interest and lifelong learning. The resources being created by the IMiLI will not only satisfy this need but also, as a single curriculum for all ages, add coherency to lifelong learning and the ability of learners of all ages and cultures to communicate and exchange information within the IMiLI education ecosystem.

*System resilience of society: increasing societal engagement in problem‐solving and provision of services*. As modern civilisation has progressed from hunter‐gatherer, via Stone Age farmer, there has been increasing specialisation of roles in relation to the division of labour. Whereas not that long ago, families were inter alia able to build dwellings, cultivate food crops in a self‐sufficient manner and prepare all food from raw ingredients, today in typical high‐income countries, we may work in an office or at home and often buy processed foods or ready‐made meals, or order delivery of ready‐to‐eat meals. If something needs maintenance or goes wrong within the home, in relation to the fabric of the building, or even in the garden, we may engage a specialist to fix it. We pay taxes and in return receive access to roads and other infrastructure, healthcare, education, protection against crime and so forth.

Major challenges confronting humanity that include global warming, unsustainable practices, the massive inequalities among peoples and the need to level up, large‐scale conflicts, pandemics, food insecurity, human migrations and organised crime, divert and consume huge amounts of economic resources (including tax revenues used to provide us with services), are raising debt levels and the financial cost of servicing this debt and driving cost of living increases. There is no indication that this situation will improve for the foreseeable future. Rather, such crises and limited intrinsic planetary‐biosphere resources and services for the world's growing human population, may worsen such problems in the future, witness the problems, such as reducing access to healthcare and deteriorating infrastructure, experienced in many countries. As a result, basic services funded by taxation that are currently taken for granted may become increasingly restricted and compel major changes in human lifestyle, behaviour and expectations.

There will be several ways and means of dealing with these challenges, but one will certainly be increased personal engagement of people in the acquisition of/securing access to products and services currently provided by specialists (either employed by the state and funded by tax revenues or directly by us), i.e., an increase in self‐help/do‐it‐yourself (DIY). Such people will also be engaged in helping others less able to engage in DIY to obtain the same services, a process that will be promoted by increased social responsibility/global citizenship of the type the IMiLI aims to encourage. The diversity of relevant products and services will be significant, ranging from food and nutrition, clean water, primary healthcare (e.g., Timmis & Timmis, 2017) and so forth. An increase in self‐help willingness and competence and resulting DIY activity, will endow greater resilience on over‐specialised modern societies vis‐à‐vis the challenges they will face. It will also result in increased empowerment of the individual‐families‐friends networks and communities, further democratisation of relevant knowledge, increasing appreciation of diversity because abilities will count more than biases, all of which will contribute to motivation.

Crucial to effective engagement in self‐help/DIY activity will be acquisition of relevant knowledge and expertise from its specialist practitioners. Many of the services that are currently inadequate in certain communities or will become inadequate as a result of the challenges on the horizon, involve microbes/microbial technologies, so literacy in relevant aspects of microbiology/microbial technology enabled by the IMiLI will be a significant facilitator of improved societal resilience.

Moreover, such societal changes will require acceptance and active participation of individuals, communities and nations and this in turn will require that people understand the causes of challenges and the potential remedies and all this in the face of pushback by vested interests and mis‐ and disinformation. It will be essential that society is properly informed about relevant issues so that it can form evidence‐based opinions that promote acceptance of necessary change. Scientists in general and microbiologists in particular, have a duty of care, a special responsibility to inform the public.

### What is the IMiLI?

**FIGURE 4:**
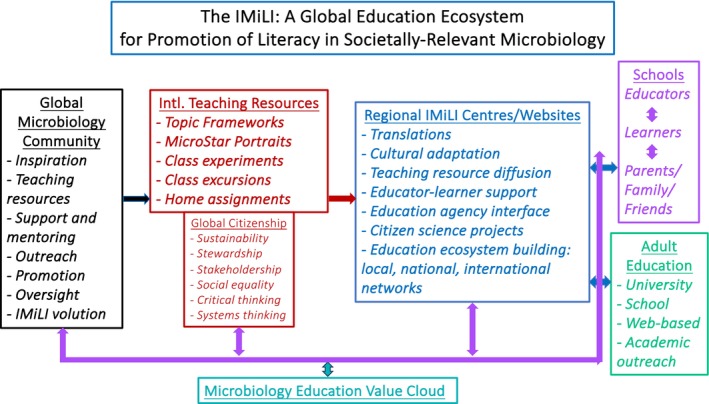
The IMiLI concept.

The IMiLI is a global microbiology education ecosystem, depicted in Figure 4, consisting of

- a *concept* of an international curriculum in societally relevant microbiology with a *vision* to enable children and adults worldwide to acquire knowledge and understanding vital to the well‐being of society and the biosphere,
- a *global community of microbiologists* constituting a platform dedicated to the creation of the teaching resources that make up the curriculum,
- the *teachers, the taught and the independent learners (the global classroom)*
- the interfacing *Regional Centres* that adapt and make available the teaching resources, provide support, create synergistic networks within and between the regions they serve.

But the IMiLI is also a *conviction* by the contributing microbiology actors, that microbial technologies can make a material difference to society, its well‐being and that of the biosphere and a *determination* to promote their increased deployment through the broadest possible exposure of their potential.

### The privilege and satisfaction of engagement in microbiology: a humanitarian calling

Saving lives and reducing human suffering are among the greatest callings of humanity, an endeavour usually considered to be primarily the realm of clinicians–the healers–although some non‐governmental aid agencies, emergency responders like the police and fire services, other health workers and carers and so forth, also enjoy this privilege. But so do microbiologists, like those involved in development of vaccines, life‐saving drugs and microbiota therapies, or in monitoring infectious diseases, but also those who develop and bring to settlements who need them technologies to microbially clean faecally contaminated water, to treat wastewater and remove toxic chemicals from drinking water, to reduce starvation and malnutrition by increasing agricultural yields. All these and other activities of microbiologists save lives and reduce disease and suffering.

But it is not only a case of saving human lives: we are part of a global ecosystem of interdependent organismal interactions whose healthy functioning requires a high diversity of participating organisms: we need to prevent the loss, that is save the lives, of other organisms. So developers of agrobiochemicals that reduce the use of chemical fertilisers which cause eutrophication and de‐oxygenation of water bodies and the killing of oxygen‐requiring aquatic animals, of biomining technologies that reduce acid mine drainage pollution, of biodegradable bioplastics that reduce the use of petro‐chemical‐based plastics and their global contamination, of all manner of technologies that reduce greenhouse gas production and the resulting global warming that is making life impossible for some species, are all in some form or other saving lives and reducing suffering. In this sense, microbiology is broadly a life‐saving and suffering‐reduction occupation. Therefore, even if these benefits are often less direct and immediate than healing by clinicians, microbiologists have the privilege and satisfaction of knowing that they make a major contribution to humanitarian efforts in society. Microbiology is indeed a great humanitarian calling and microbiologists have a duty to reveal this to children!

### ‘Tries hard but could do better’: democratisation of microbiology and learner‐driven evolution of the IMiLI

It is important to emphasise that although the current IMiLI teaching resources being released–the TFs and Gallery Portraits–in our view constitute a critical mass, they are far from complete. The curve of resource additions is asymptotic: it is likely that the plateau will be approached sometime in 2026, but further additions will accrue thereafter. So there is still much to be done and the IMiLI welcomes offers to get involved and contribute teaching resources.

Moreover, we do not pretend that the resources currently being created represent the best that can be devised: this is the initial iteration. Our notions of child/student‐centricity and what is societally relevant are certainly incomplete and the reader, end users of our resources and others, will have additional ideas, ideas that can flow into the dynamic evolution of the resources we envision. This is particularly true for changes that reflect cultural needs. Importantly, democratisation success will depend not only on creating teaching resources that fascinate learners and for which they provide positive feedback, but also stimulating them to explore and discover new topics that excite them and for which the IMiLI should create new teaching resources. Inherent in the exercise of creating new teaching resources will be a significant degree of autocorrection: authors will read their previous contributions and those of others and note mistakes/improvements that they hopefully will communicate to the IMiLI for correction.

**FIGURE 5:**
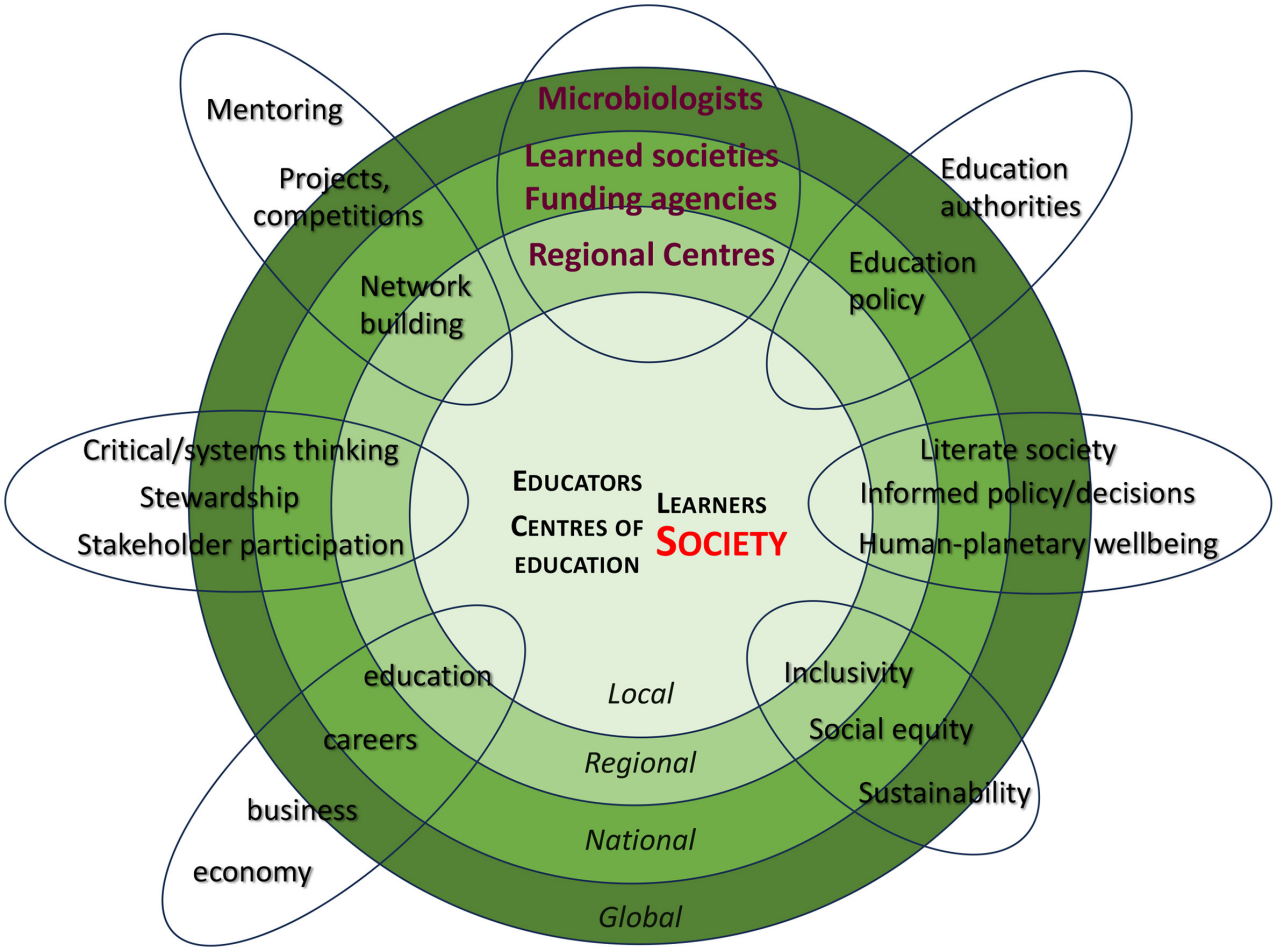
Principal actors in and drivers of evolution of the IMiLI.

Although ad hoc updating will occur continuously, at some point an IMiLI 2.0 will be needed that will add new materials to reflect changes in local and global situations, improve or delete some of the less useful materials and especially that will respond systematically to suggestions and critiques of educators, children and other end users (e.g., see Figure 5). Current formats may change. This will undoubtedly result in a revised and superior collection of teaching resources co‐created by creators–educators–learners. The current creators of the teaching resources–the hundreds of microbiologists, clinicians, ecologists, biochemists, immunologists, bioinformaticians, bioengineers and more, from all over the world–are also learners! Importantly, creators, educators and the taught are a single, interactive ecosystem that will naturally continue to seek to achieve the best solution for all. As Dieter Czeschlik of Springer Verlag once remarked to KT during a discussion about a handbook series: ‘the second edition is always significantly better than the first’! But, for the reasons stated above, we suspect that the teaching resources of IMiLI 1.0 are ground‐breaking and pioneering and therefore will have a special value for the first generation of teacher–learners who use them to learn societally relevant microbiology.

## AUTHOR CONTRIBUTIONS

Kenneth Timmis created the various drafts of the manuscript. The other authors provided additional ideas and suggestions for improvements.

## Supporting information


Appendix S1.
