# Supplementary material for: A concept for international societally relevant microbiology education and microbiology knowledge promulgation in society
Source: Microb Biotechnol. 2024 May 27;17(5):e14456. doi: 10.1111/1751-7915.14456 (PMC11129164; doi:10.1111/1751-7915.14456)
Supplement: Supplementary file 1 — Appendix S1. [file MBT2-17-e14456-s001.docx]

**Supplementary Material**

**Data S1: Examples of impacts of microbial activities on everyday life and relevance to informed decision-making**

| **Issue: personal; daily** | **Potential impact** |
| --- | --- |
|  |  |
| What food to eat (e.g., meat, fermented food, food from organic or conventional production)? | GHG production and global warming; environmental pollution; eutrophication; antimicrobial resistance; human health |
| What type of clothing to wear (e.g., shirt/blouse made of cotton, polylactate, polyester)? | Production footprint (water, environment); life cycle (rapid recycling vs. long life in landfills) |
| Degree of ventilation of homes/workplaces/other enclosed spaces? | Increased ventilation results in reduced transmission of respiratory infections; reduced growth of mould on surfaces; increased microbial diversity of living space/occupiers; decreased levels of volatiles released from heating/cooking processes, household materials |
| Frequency and mode of taking care of our teeth? | Impact on caries and periodontal disease; suspected impact on chronic diseases like cardiovascular disease, diabetes, pancreatic cancer |
| Smoking, vaping | Impact on airway microbiota and the services they provide |
| Use of biodegradable plastics that can be composted at home or commercially | Reduction of plastic pollution |
|  |  |
| **Issue: personal; regularly** | **Potential impact** |
|  |  |
| Kitchen practices? | Food contamination/food poisoning |
| Degree of humidification/dehumidification of homes, workplaces, other enclosed spaces? | Increased humidity is good for mucosal surfaces, but also promotes mould growth; increased risk of dry rot in buildings |
| Frequency of use of strong disinfectants to clean the home? | Reduced microbial diversity of home/family; effects on aquatic life |
| Use of phosphorus-containing household cleaning products? | P contributes to eutrophication; is a limiting resource; increased P-mining activity |
| Regular use of germicidal hand washes? | Change, perhaps long-term, of hand microbiota; input of antimicrobials into aquatic environments |
| Use of germicidal soaps? | Perturbation of the skin microbiome and its barrier function; effects on aquatic life |
|  |  |
| **Issue: personal; variably** | **Potential impact** |
|  |  |
| Vaccination? | Prophylaxis or therapy? Reduction in transmission; reduction in days lost at school/work |
| Antibiotic use? | Reduction in microbiome diversity, the services it provides, and its barrier function; increase in risk of spread of antibiotic resistance |
| Sexual behaviour? | Sexually-transmitted infections |
| Caesarean (aseptic) or natural delivery birth? | Maternal-baby microbiome transfer |
| Breast- or formula-feeding infants? | Human milk oligosaccharides structure developing gut microbiome, which influences immune system development; potential disruption to daily routine of mothers |
| Greeting modes involving hugging, kissing, shaking hands? | Positive consequences: enrichment of the human microbiome; negative consequences: increased exposure to respiratory and oral-faecal infections |
| Acquire a companion dog? | Increases microbial diversity of the home/family/child; zoonotic infections; dogfood production footprints: greenhouse gases, N/P and eutrophication, agrochemical pollution, competition for agricultural resources and impact on food security |
| Live in urban or rural settings? | Urban life involves reduced microbial diversity and air pollution negatively impacts airway health, especially of children; rural life is associated with high microbial diversity and low air pollution |
| Travel | Exposure to different types of food-borne/water-borne and respiratory infections; dispersal of microbes; carbon footprint and climate change |
| Swimming | Water-borne infections |
| Camping, activities in vegetation-rich habitats | Increased exposure to vector-borne infections |
| Tattooing? | Potential skin infections |
|  |  |
| **Issue: communal/national** | **Potential impact** |
|  |  |
| Various activities associated with wastewater treatment | Lowers environmental footprint of wastewater; elimination of pathogens; removal of toxic chemicals; provides opportunities to recover and recycle N and P; provides opportunities for community pathogen surveillance |
| Renaturing grasslands in cities | Increased plant and animal diversity, impact on microbiota composition, air quality |
| Deforestation | Increased soil temperature; decrease in beneficial microbial activities, including carbon sequestration |
| Land sealing | Prevention of soil vitalisation and carbon sequestration by plant:microbe partnerships; effects on GHG fluxes and global warming; reduced land use and threat to food security |
| Farming practices: use of agrochemicals | Modification of soil and plant microbiomes, soil health and fertility and thus to food security; negative effects of run-off on aquatic life |
| Farming practices: antibiotic use in animal food production systems | Modification of animal-associated microbial communities, emergence and spread of antibiotic resistances |
| Farming practices: soil compacting | Reduced soil microbial diversity |
| Fossil fuel burning | CO_2_ release and global warming; increased concentrations of CO_2_ in aquatic and marine ecosystems; destabilization of carbonate minerals due to acidification |
| Hard rock mining | Increased production of acid mine drainage due to microbial activity, and effects on aquatic life |

Abbreviations GHG: greenhouse gas; N: nitrogen; P: phosphorus

**Data S2: Instructions given to authors creating teaching resources**

| **DO** | **DON’T** |
| --- | --- |
| Explain key messages to educators in easy-to-understand language so that they can relay this information at whatever level is appropriate to their class | Make it comprehensive - leave that for later education, once the passion for knowledge has taken root |
| Make it learner-centric: relevant to their current experiences and preoccupations | Make it complicated |
| Make it visual and colourful | Use technical language and terminology (employ language understandable by the general public) |
| Have it appeal to children’s thirst for exploration and discovery |  |
| Reveal the wider context and the connectedness of everything, *inter alia* by interweaving information from past and future lessons |  |
| Make it an enabler of better adulthood |  |

**Data S3: Food – microbial control of operational space twixt bedrock/seabed and sky: an example of the pervasive interconnectedness of things**

*Soil: plant food and support.* Plants in general, and food crops in particular, mostly grow in soil which provides not only anchorage for plant roots, but also water and minerals such as nitrogen, phosphorus, and sulfur that plants need to build cells and grow. Some microbes disassemble and dissolve rocks, creating the soil that plants need to root in, and release minerals essential for plant growth. Some soil microbes transform atmospheric nitrogen into ammonia, and thereby provide plants with a key element needed for growth. Yet others transform phosphorus – another element essential for plant growth – which is largely present in soil in a form inaccessible to plants, into forms that are accessible. Yet other microbes produce plant-like hormones that promote plant root growth and thereby facilitate plant acquisition of nutrients. As a result of the microbial gifts of these fertilisers and other compounds, plants grow and, in return, channel photosynthetically-produced carbohydrates into the soil that feed soil microbes. These in turn grow and create much of the soil organic matter (SOM), which represents a sink for the carbon dioxide (CO_2_) fixed by the plants through their photosynthetic/photoautotrophic activity, and an important component of soil fertility. Plant-associated microbes are thus key players in carbon fluxes, carbon budgets, greenhouse gases (GHGs) and global warming, and soil fertility.

Soil properties vary according to composition and climate, the latter aspect becoming increasingly relevant at a time of accelerating climate change. Areas with low rainfall have arid soils with poor plant vegetation coverage and thus poor microbial productivity and fertility. Nevertheless, soil microbes respond to water deficiency stresses by forming photosynthetic biocrusts on the soil surfaces that stabilize the underlying soil matrix, retain water and increase organic matter inputs, thereby improving soil quality and enabling plant growth above what would otherwise be possible.

*Plant microbiomes: services provided.* Like all organisms of the biosphere, plants have microbiomes – the microbes that form a second skin covering roots (rhizosphere), leaves (phyllosphere) and stems, and that live within plant tissues (endosymbionts). Plant microbiomes provide services essential to the host plant: reducing infections and parasite attacks, providing essential chemicals and hormones, and increasing resilience to stressors such as desiccation. But there are also microbial pathogens that cause plant morbidity and mortality. Microbes therefore are crucial determinants of plant health and, in the case of crop plants, their yields and quality, and hence of food security.

*Upgrading plant food.* Plants and plant products serve as a source of food for herbivores and omnivores, such as our food animals, which are in essence powerful biomachines that convert low protein plant materials into protein-rich meat or dairy food. Central and essential to this process are the agricultural animal gut microbes which digest plant materials, many of which are otherwise not digestible by the host animal itself, and in the process deliver to the host digestible fuel and food, proteins, and essential growth factors, like vitamins. Ruminants, like cows, sheep, goats, and camels, are a special example of herbivores that possess a rumen, an extra component of the digestive tract, which is essentially a chamber containing microbes that transform renewable but poor nutritional quality, low protein grass, hay and straw into high quality, protein-rich food like meat and milk for humans. In addition, some of the rumen microbes remove toxic compounds produced during digestion of grass and hay, such as acetone which they transform into succinate.

*Carbon footprint of food systems.* However, the rumen is an anaerobic digester containing special types of microbes that utilize specific molecules and metabolites generated in the digestion process and that can convert them to methane (CH_4_) – *a lot of methane* – a greenhouse gas (GHG) which is exhaled to the atmosphere. Food production systems collectively contribute a quarter of all human-generated carbon emissions, and beef production has more than twice the carbon footprint of any other food (<https://interactive.carbonbrief.org/what-is-the-climate-impact-of-eating-meat-and-dairy/>). Alternative food sources that produce less methane are urgently needed to mitigate food-related greenhouse gas footprints (see below). However, in many impoverished regions of the world, ruminants will continue to provide valuable sources of nutrition and income into the future, so microbiology-based technologies that reduce methane from ruminants are of upmost importance.

*Alternative microbial food sources.* Since food animals, especially the ruminants, are associated with high carbon footprints, non-animal nutrition products are growing in popularity. While plants and mushrooms continue to be the mainstay of vegetarian diets, other microbial foods and nutrition products are gaining traction. These include fungi (mycoprotein), photosynthetic cyanobacteria and microalgae, like *Chlorella,* which can be processed into a variety of dietary supplements or whole foods, some of which are marketed as meat substitutes. Then there is the effort to create “true” and “authentic” cooked meat flavours and colours, one of which involves the microbial production of haemoglobin that can be added to vegetarian products.

*New food for old.* Once our plant and animal food has been harvested, it may be transformed to upgrade (e.g., cheeses), extend its shelf-life (e.g., fermented foods) or create entirely new forms (e.g., bread, alcoholic beverages, etc). Chocolate, coffee, table olives, various sauces, and condiments, like soy sauce, all involve microbial fermentations in their preparation. Some fermented products, such as yoghurt, are being developed as health products that positively influence the composition of the gut microbiota or provide beneficial metabolites. Microbial transformers are at the heart of the quality and diversity of our diet.

We also ferment food for our farm animals for exactly the same reasons we ferment food for ourselves, specifically to increase nutritional quality, diversity and shelf life. This is the process of ensilaging, the fermentation of green plant material, such as grass, hay, or corn stalks, and often involves microbes related to those used in human food fermentations.

*Food supplements*. Because the food eaten by some people does not contain individual amino acids in the required amounts, their diets need to be supplemented. This is also true of food animals. Amino acids are made to a significant extent biotechnologically, and are produced by microbial cell factories, transforming sugars to amino acids.

*Food spoilage and quality control.* But of course, microbes can also spoil food: like us, they like to eat and when they eat *our* food, they can transform it into a less palatable form that looks, smells, and tastes bad (of course they can also turn it into something healthy and delicious: the fermented foods). They can even make food dangerous: pathogens can infect us, and toxin-producing microbes (e.g., enterotoxin-producing staphylococci, mycotoxin-producing fungi) can poison us. Food quality control is thus important and newer methods involve detection of viable pathogens by reverse transcription polymerase chain reaction (RT-PCR) using *inter alia* the microbial Taq polymerase.

*Preparing food for a meal.* The process of obtaining food materials and preparing a meal varies enormously from essentially no effort, as in microwaving a frozen TV dinner/picking up or having delivered a ready-made meal, to great effort, as in foraging-hunting for food, and/or spending the entire day preparing and cooking it, with everything in between. In some instances, the preparation of food is done by one person, whereas in others, it involves more than one person, sometimes with a strong element of social interaction with family/friends/neighbours working together, so includes interpersonal bonding with all the social and mental health benefits that arise from this. Although the process of food preparation is mostly non-microbial in purpose, one essential aspect is the elimination of potentially dangerous microbes. This is by washing, for example vegetables and fruit that will be consumed raw, the plants of which may have been fertilised with pathogen-containing animal or human wastes, and by cooking, for example removal of enteric zoonotic pathogens like *Salmonella* and *Campylobacter* that often contaminate poultry and eggs, and pathogens that may be present on vegetables. In addition to the possibility that food entering the kitchen is already contaminated by pathogenic microbes, it is also possible that food materials may become contaminated in the kitchen itself by a person involved in meal preparation. This is particularly important in restaurants because of the potential to infect large numbers of people. Proper hygiene practices and cooking are key to preventing transmission of pathogens from persons preparing food to those consuming it.

*The meal.* Then there is the process of food consumption, the meal, which again varies considerably in manner, time, and social context. But, in most societies, eating together is a key event of the day that may contain a multitude of social aspects, including family bonding, interaction with friends and neighbours, leisure/relaxation, information transfer/exchanges (including touching base over how the day went), instruction/education, and so on, again some of which contribute to mental wellbeing. And, depending on who is eating with whom, these social aspects may also involve romance, politics, business, and contributions to other inter-personal activities. Meals may also be accompanied by the consumption of fermented drinks created by microorganisms, such as lassi and other yoghurt drinks, and alcoholic drinks which may contribute to conviviality of the occasion. (Microbes influence mental wellbeing, as do social interactions associated with food preparation and consumption, so it would not be surprising if there is interplay between the human microbiome, the type of food consumed, and the degree of social interactions involving food. But, at the time of writing, investigation of these possible interactions is still in its infancy.)

*Food digestion.* The purpose of meals is to supply our bodies with fuel and essential materials needed for our growth and activities, proper functioning of our bodies, and repair of the daily damage that is caused to our cells/tissues. Because food mostly consists of complex polymeric compounds that cannot be absorbed by our intestines, food digestion involves its deconstruction – breakdown – by digestive enzymes into its component building blocks that can be absorbed and further metabolised by our cells. However, many of the components of our food cannot be deconstructed by our own digestive enzymes. What is more, some of the substances we require for our health, like some vitamins, cannot be manufactured at all by the human body itself, no matter what we eat. So, once we have had an enjoyable meal, the microbes in our gastro-intestinal tract take charge, helping us to digest the food components which feed both us and them, and producing substances that we need but cannot produce ourselves.

Our gut microbes also transform some food components and their breakdown products into a vast range of chemicals, some of which are essential for us and others of which have hormonal (so act in effect as our second endocrine system) or physiological activities that influence our health (mental and physical wellbeing), emotions, moods, and hence behaviour. Since what we eat influences the composition, and hence activities of our gut microbiota, our meals not only have major *direct* consequences for our wellbeing, but also *indirect* microbially-mediated consequences for our wellbeing. The motto *you are what you eat* thus has two strands, that not involving the microbiome and that dependent on the microbiome. But the two strands are tightly interwoven! What we eat modulates the composition and activities of the gut microbiota and our physiological and metabolic relationship with it and can impact the onset and progression of chronic diseases such as obesity and diabetes. This is especially relevant for individuals whose diet lacks a healthy content of non-processed foods including fruit, vegetables, grains, nuts, pulses, fibre, etc. Taking into consideration foods that promote the growth of beneficial microbes as part of meal planning meals is becoming an increasingly important aspect of healthy living, and of so-called *precision* (personalised) *nutrition*.

*Treatment of what comes out the other end.* And once our bodies have finished with the food, we dispose of what remains and many of the microbes that have been produced during the process of digestion (about 50% of faeces is food remains and the other 50% is microbes) into the toilet via our faeces, which are then transported to wastewater treatments plants. Here, they are taken in charge by yet other microbes that transform them into biomass and recycle them into the end products of metabolism, carbon dioxide and methane. However, wastewater treatment is increasingly focusing on recovering rather than destroying the components of sewage, both to retrieve valuable resources, such as nitrogen and phosphorus in forms that can be used by plants, and advance towards a circular economy.

*Antimicrobial Resistance (AMR).* Antibiotics in clinical use, most of which are inspired by inhibitors produced by microbes to defend themselves against competitors, are wonder drugs that have saved innumerable lives and constitute one of the key components of the pharmaceutical arsenal of modern medicine. They are essential for the treatment of severe bacterial infections and prevention of infections during and following many surgical interventions. Antibiotics have also been, and in some countries continue to be, massively used in animal husbandry and aquaculture to reduce microbial infections and attain higher growth rates/yields of food animals (refs). They are also used to a lesser extent for plant crops, like rice and fruits (see e.g., Taylor and Reeder, 2020). Since this selects for microbiomes that are resistant to the applied antibiotics, antimicrobial resistant (AMR) microbes are transmitted to anything and anyone in direct contact with the plant crops, food animals and their products. These contacts include farm personnel, farm soils, surfaces and equipment, and the animal products and the personnel handling the animal products in the abattoirs and butchers. There are also indirect contacts, such as scavengers (like vermin, birds, foxes, and insects), personnel servicing farm equipment or transporting farm products and wastes, which in turn can transmit the microbes to others.

Animal wastes are a particularly rich source of AMR microbes and the routes that wastes travel provide further opportunities for transmission to other people and environments. Some wastes also end up in the sewerage system which transports them to wastewater treatment plants, which are not only rich sources of resistant microbes but also hotspots for transfer of AMR genes to new microbes. Other hotspots for AMR gene transfer are soils, which are also teeming with microbes, especially those directly receiving untreated faecal material (5% of the world’s population defaecate in the open: https://www.who.int/news-room/fact-sheets/detail/sanitation) and wastewater (much of the world’s wastewater is not treated (https://www.who.int/news-room/fact-sheets/detail/sanitation), but simply dumped onto the earth).

Some AMR microbes of farm animals are pathogens for those animals, and some are also pathogens for humans, *and vice versa*, and so represent treatment challenges in veterinary and human medicine. More importantly, however, the AMR microbes of farm animals can transfer their resistance genes to other microbes, including human and veterinary pathogens, which then become difficult to treat. Furthermore, several commensal bacteria – bacteria that are normal, usually harmless inhabitants of our bodies – that have become resistant to antibiotics can become life threatening if they are introduced into the blood stream during surgery or by an injury. AMR is considered by the WHO to be one of the most serious threats to human medicine, and the O’Neill Report

(https://amr-review.org/sites/default/files/160525_Final%20paper_with%20cover.pdf) forecasts that by 2050 it will be responsible for 100 million deaths annually, thereby becoming the number one cause of death, and a cumulative loss of economic output worth US$ 100 trillion.

Importantly, antibiotics and their metabolic products originating from clinical and non-clinical use, and from antibiotic manufacturing facilities, are pervasive environmental pollutants, often present at low concentrations that are nevertheless significant in terms of biological activity and impact. Fortunately, microbes have evolved to exploit almost any source of energy and carbon, including antibiotics, and so eventually most antibiotics ending up in the environment are likely to decompose and be consumed. The problem is that the rate of environmental input appears to be higher than the rate of microbial consumption, which ensures the presence of antibiotics in surface water supplies. These in turn represent a powerful and persistent source of pressure for microbes to retain and transmit antimicrobial resistance among other environmental microbes. This antibiotic abundance also causes perturbation of ecological food webs and the microbiomes of plants, animals, and humans.

*Concentration and mobilisation of nitrogen and phosphorus: eutrophication and oxygen-minimum “dead” zones, global warming, and a planetary tipping point.* The entire chain of food production not only involves microbial transformations that ultimately feed us and allow us to grow, but also bioconcentration of nitrogen (N) and phosphorus (P) (some originating from microbial activities, some from the agrochemical industries) in our bodies and wastes, and those of farm animals. Some of this N and P cycles back into soils and waters and adds to the N and P applied as fertilizer to promote plant growth. However, not all is used by plants, and some is washed into local water bodies. Here, it can promote the growth of resident photosynthetic cyanobacteria and microalgae, the blooming of which causes eutrophication of water bodies which, in turn, leads to rapid consumption of oxygen by heterotrophic microflora and the creation of oxygen-minimum zones that do not support oxygen-requiring multicellular forms of life. This exacerbates the loss of biodiversity. N-wastes can be recycled back to nitrogen gas, but biological processes are rarely 100% effective, and so some is only partially transformed and ends up as nitrous oxide (N_2_O), a powerful GHG. The concentration and mobilisation of biologically available nitrogen, its creation of eutrophication and oxygen minimum dead zones in aquatic systems, and conversion to nitrous oxide which contributes to global warming, constitute the nitrogen crisis, one of the planetary tipping points (Rockström, et al., 2009; Canfield et al., 2010; Rucker and Kaçar, 2023).

While nitrogen fertiliser is created from an unlimited supply of nitrogen gas, phosphorus is a natural mineral resource obtained by mining. Sources of phosphorus are limited, and its exploitation largely involves a linear path from mine to ocean, which is clearly unsustainable. The challenge is to develop a circular P-economy and in this regard major progress is being made at the level of wastewater treatment plants.

*Waste recycling.* The production and consumption of food are accompanied by the production of considerable wastes and by-products. Some of these can be fed to farm animals, others may be composted, and yet others may end up in landfills. In all cases, microbes digest and recycle them. Recently developed processes embedded in wastewater treatment plants use microbial activities to recover N and P in the wastes, thereby preventing escape into the environment, and enabling their re-use as agricultural fertilisers. Dedicated microbial P-collectors will in addition support the transition to a circular economy, upcycling P to polyP. Such processes will enable significant reductions in the massive amounts of energy currently used to produce fertilisers (1-2% of total global energy expenditures), and greatly improve the security of fertiliser supply.

*Pollution mitigation*. Some agrochemicals, especially some pesticides and herbicides, are environmental pollutants of concern. Moreover, the use of antibiotics to treat farm animal (and human) infections and, in some countries, as growth promoters in animal husbandry and aquaculture, results in the release of huge amounts of antibiotics into the environment, which should also be considered as agrochemical pollutants. As the world’s population grows, and food demand increases, so too do agrochemical use and environmental pollution. But then: microbes to the rescue (at least in part)! This is because microbes evolve and share the ability to degrade and destroy agrochemicals, including antibiotics, pesticides, and herbicides, sometimes using them as microbial food. Microbes are the environmental cleaners of the food industry.

*The microbial methane filter.* Much of the organic wastes, including food waste, ends up in landfills, mostly under anaerobic conditions and hence are recycled by anaerobic microbes to methane, an important greenhouse gas. However, as the methane migrates upwards to landfill surfaces, it encounters oxic conditions – regions containing oxygen – and the methano/methylotrophic microbes that live in such regions and use methane as food. These methano-/methylotrophs constitute the *methane filter* and prevent much of the methane produced in landfills from escaping into the atmosphere and contributing to global warming.

*Bioenergy.* Animal husbandry wastes and sludge from wastewater treatment plants are collected in anaerobic digesters where microbes recycle them to form methane, a valuable source of bioenergy which is used to power electricity generators. Similarly, methane from landfills and other anaerobic facilities is collected for use as an energy source. Wastes are also being used to power bioelectrical systems, such as microbial fuel cells, which are being developed as electrical energy sources and batteries. One application of these is in desalination, the removal of salt from seawater and brines to create freshwater. Thus, the energetic resources of wastes are being captured and exploited in diverse ways, thus contributing to energy security.

In the near future, methane from these and other sources will also be converted into microbial biomass that can be used for diverse purposes, including food (single cell protein) for direct human consumption (see *Alternative microbial food sources* above) or indirect consumption, by using as food for farm animals, in both cases thereby reducing agricultural land use.

*Economy*. Many enterprises and employment opportunities are based on or intimately bound up with microbes and their activities relating to food production. These include:

- the production, quality control and sale of microbial seed inoculants that increase nitrogen fixation in the plant roots or rhizosphere, phosphorus solubilisation/mobilisation, plant immune responses to pathogen attack, plant growth rates, plant tolerance of stresses, and that inhibit pathogens.
- the upgrading and diversification of food products by microbial activities/fermentation, such as the production of fermented milk products and cheeses, the manufacture of the exceptional range of cheeses of all manner of flavour, aroma, consistency, and appearance through maturation with other microbes, the production of diverse breads and sourdough breads, beers, wines and other alcoholic beverages, fermented sausages, chocolate, coffee, table olives, pickles, sauerkraut, and many other foods.
- the production, quality control and sale of microbial inoculants for food fermentations, especially yeast to produce bread, beer and wine (yeast extract is of course also used as a dietary supplement and for food flavouring), and bacterial starter cultures for yoghurt and kefir production.
- microbial production of food supplements (vitamins, amino acids, sterols, etc.)


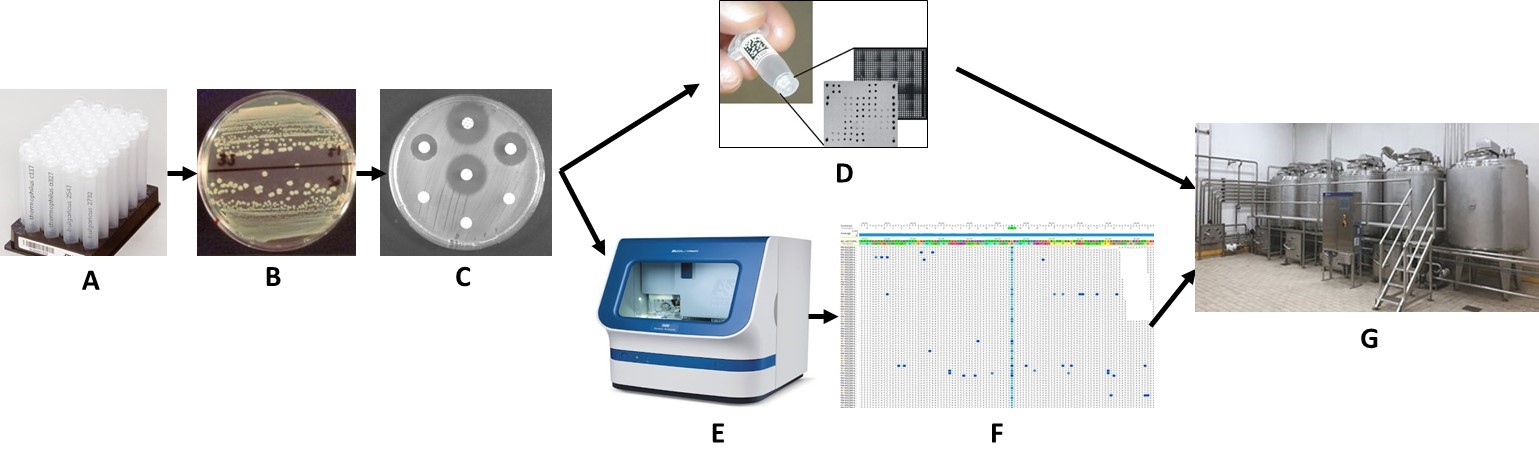


**Analysis of yoghurt bacteria for absence of antimicrobial resistance genes.**

A) Yoghurt producers have collected a large number of frozen bacterial strains of *Lactobacillus bulgaricus,* and *Streptococcus thermophiles* selected traditionally by their properties of vitality and suitability for producing yoghurt of various tastes. B) Before using them in production, strains are grown and tested for purity. Single colonies as shown on a bacterial growth plate must be uniform. C). To ensure that these bacteria do not harbour Antimicrobial Resistance (AMR) genes, they are first tested by the classical antibiotic inhibition zone test. The plate contains a lawn of the bacterium to be tested. Each white circle on the plate is a small filter paper disc impregnated with a different antibiotic. Transparent halos around the discs indicate that the growth of the bacterium is inhibited and accordingly the bacterium is sensitive to the corresponding antibiotic. New methods have been developed to further detect potentially ‘silent’ AMR genes: D) DNA of the bacterium was extracted and analyzed on gene microchips that detect all known antibiotic resistance genes (black points), or E) DNA of the bacterium is fully sequenced and F) the millions of base pairs are analyzed by bioinformatics and adaptive algorithms for presence respectively absence of antibiotic resistance genes. G) Strains that pass the quality tests are then used in industrial yoghurt fermentation to insure a safe and healthy food product.

*Social and socio-economic components of the network.* Food preparation and consumption are also dominant influences on our lives and wellbeing. Food sourcing and preparation, sometimes alone, sometimes as a group effort, can be on one hand a burden, contributing to life’s stresses and negatively impacting physical and mental health, but can also be enjoyable, positively impacting wellbeing and, if in a group, contributing to familial and social cohesion. In some families/cultures, it takes up a major part of the day, so constitutes a significant element of life’s activities. For practicing gourmets, food sourcing and preparation is a major source of pleasure and satisfaction. And, as indicated above, food consumption is, for many people, a crucial social event that serves multiple purposes, such as family-friend bonding, information exchange-education-upbringing/parenting, business transactions, and planning and strategic exercises, just to name a few.

From this, it can be seen that food production, consumption and downstream consequences involve a broad range of connected and interdependent processes that range from soil production and fertility, via food plant growth and ecophysiology, food animal husbandry, land use-ecosystem services provided by soils-food security agrochemicals production and pollution of soils and water bodies, eutrophication-oxygen minimum zones-killing of marine wildlife, reductions in biodiversity, energy inputs and outputs, production of greenhouse gases and climate change, food conversions, nutritional imbalances and food supplements, food safety and food-borne infections, microbiomes and their importance for crop plant, food animal and human wellbeing, waste treatment, economics and employment, to social interactions. Food systems are globally connected via supply chains, pollution via freshwater and marine systems, greenhouse gases and their effects on climate, and so forth. Crucially, microbes drive or are involved in almost all of these processes and are thereby the bionodes and biolinks of a highly interconnected and interdependent network of diverse global processes. And they are at the heart of a large number of intertwined crises facing us now and in future, either as contributors to, or mitigators of, the problem, or as potential elements of solutions (see Timmis et al, 2019, for a detailed discussion).

**Food interdependencies web**

In addition to illustrating some of the microbially-centric interdependencies associated with food production, consumption and waste treatment, this figure, in depicting the relationships between human, animal, plant and environmental healthcare in the food chain, also mirrors a number of elements of One Health (<https://www.avma.org/sites/default/files/resources/onehealth_final.pdf>; <https://www.fao.org/3/cc2289en/cc2289en.pdf>).

**Data S4 Telling comments**

The large number of authors of this Editorial contributed many ideas and suggestions for content. While a lot of of these could be incorporated in some form into the final text, others could not without prejudicing coherence. But some of them are priceless gems/words of wisdom and absolutely belong to this piece, so are reproduced below.

***Rachel Armstrong***

Storytelling for the 21st century: microbes, through the toolsets provided by the IMiLI, go beyond education as such and become a creative catalyst for retelling the story of our world and our role within it. We need EPIC stories of the world, not only the ones that are rooted in the present dominant frameworks centred on human consciousness, but that engage with the life of our world. e.g. a story of humanity exists through our 'evolutionary path' from ancient microbial ancestors. This helps us see the similarities between us and microbes and understand our interdependencies through commonality.

More than human ethics: an environmental ethics could be positioned within the portfolio. Microbes provide a tangible way of being good environmental citizens, which is not unproblematic, but it invites a kind of microbial permaculture as a practice. In this way we can learn through microbial ethics and associated rituals of waste processing, environmental stewardship, personal care, probiotic hygiene, and the like, how to be better world-citizens.

The language could be expanded from the scientific explanation which presently dominates. I wonder if there is some space for new materialist rhetoric. Perhaps it might also be an idea to include in the IMiLI a section with contributions from the Social Sciences and Humanities, philosophy and creative writing. It would be necessary to frame it in a particular way to keep the overall coherent, but to change the language for storytelling beyond accessible narratives laden with facts and to delve into experience, would perhaps appeal to some of those students that are not scientifically minded – perhaps some ‘use and experience’ stories and analyses, e.g. what does it mean to be human at a time of the microbiome? What is an environmental ethics, and how can microbes help us be better earth-citizens?

***Eric S. Boyd***

I spent yesterday getting my syllabus ready for my microbial ecology class and, after reading this, feel the need to revise it significantly to better place it within what I believe will be a transformative era of microbiology education.

***Martin Kaltenpoth***

One goal that is in my view worth adding to the list of IMiLI goals is the understanding of the scientific process itself. Especially during the pandemic, we have come to realize how important it is to educate the general public (and especially the children, as well as policy makers) how science works. Many people did not understand why scientists said one thing and then something else a week later when new data were available. I think it would be great if we could also attempt to make a contribution towards educating the public in the scientific process per se.

It would be good to highlight that the IMiLI components are brought together in a major community effort. It will be exciting for teachers and children to realize that a large international community of scientists, all individually named, agrees that microbiology literacy is very important, and that many – hundreds or perhaps even close to a thousand – made an effort to contribute, which gives the IMiLI its diversity of styles, content and emphasis.

***Oleg Kotsyurbenko:***

We are witnessing the deterioration of the global ecology. Microbiology must be among the priority topics brought to the attention of the public: we neglect it at our peril!

According to system principle, in order for the system to acquire a system property, it is necessary to establish the correct interactions of its elements and its own interactions with other systems. The concept of the triangle of knowledge involves the interaction of education, science and practice where education ecosystem is an element of a system of a higher level. The interaction in triangle of knowledge is a condition for the sustainable development of all three its ecosystems including a microbiological education ecosystem and forms a single microbiologically competent space. Important properties of such a system are the connection with modern world problems, an adaptive learning system, multi-level complexity and, consequently, a large coverage by age categories, internationality, and accessibility. In general, the principles of creating such a microbiological education ecosystem can form the basis for the development of other disciplinary ecosystems.

***Terry J. McGenity***

(Commenting on the 3 D’s) Given that the repeat of the same word has been used once already with the 3Rs, how about "deliberating, discarding* and decisioning", e.g. deliberate about diverse possible actions / brainstorm, discard those that are not (currently) feasible, and then decide (but with plan B and C)? The middle D could also be "developing" - i.e. the process of reaching a decision, which would include discarding some ideas, but which is perhaps a more positive way of framing it.

***Allen Y. Mswaka***

In some communities, especially those practising subsistence farming, children actively participate in agricultural activities from pre-school age and therefore early awareness of microbiology will help them to understand soil ecosystems and their importance in improving crop yields. These communities are typically resource-constrained and schools in such areas are inadequately funded with little or no information technology (IT). The IMiLI will enable children in these communities to acquire knowledge on the practical applications of microbiology in agriculture. Once the children have learned to apply concepts of microbiology within their local environment, this knowledge will stay with them into adulthood, creating a pool of knowledge that can be propagated horizontally and also vertically by cultural transmission. Another benefit to the community will be the strengthening of the local education ecosystem by fostering links between schools and their local communities.

In the semi-arid regions of Sub-Saharan Africa, the use of a low-cost nitrogen fixing rhizobia technology for legume crops by small-scale farmers has resulted in increased crop yield (93–102%), while at the same time reducing the need for expensive fertilizer input. Inherent in this process are ecological and economic benefits in addition to food and nutrition security.

Enable practical microbiology teaching in resource-limited communities by providing schools with free laboratory equipment through charity organisations that deliver donated lab equipment, lab supplies, and training to communities in need around the world, diverting from landfill waste.

***W. Donald R. Pokatong***

In the Indonesian curriculum for Grades 1 to 6 (Elementary School), 7 to 9 (Junior High School), there are no specific contents about microbiology. At Senior high, Grade 10 to 12, there is a biology course, and starting at Grade 10 there is a topic of Virus and Its Roles. With this Indonesian context that is lacking microbiology, I think the IMiLI has a big role to play.

***James I. Prosser***

We emphasise the importance of teaching about microbial technologies and their potential for solving societal problems, but we also need to *emphasise the consequences of ignorance.* This has been highlighted by the pandemic (microbial dispersal, epidemiology, vaccines) but there are other examples – e.g. lack of rational thinking with regard to use of genetically modified organisms, CRISPA debate, etc.

There is also a training aspect. The lack of awareness of microbiology among schoolchildren reduces the quantity and quality of those wanting to pursue a career in microbiology.

***Jyoti Prakash Tamang***

We need to develop the programme to ignite the young minds of children when they start speaking and learning to know why their mothers breast-feed – the importance of human (mother milk), why are they being fed with oats/cereal porridges, breads, rice or cooked foods, fruits, and why they have to take bath and wash hand, feet, wear clean clothes, etc. Why grandma/pa, mother/father pour water on plants in gardens, and manuring, questions that should be posed and answered so that the active memory cells of children will store and save such information for their whole lives. This is path to get children more acquainted with climate change and creating a caring attitude towards protection and conservation of the bioresources of our ecosystem. The teaching resources on fermented food section is an excellent example of how our children should start liking cultural foods coupled with scientific information on health benefits and beneficial microbes.

***Kenneth Timmis***

One motivation of the teaching resource creators is privilege: not everyone can contribute to the education of their own children/grandchildren/children of relatives and friends, and influence content and quality of teaching resources.

Learning goals (e.g. see https://evals.stanford.edu/end-term-feedback/how-write-learning-goals) seem to be *de rigueur* for most teaching courses, but often give the impression of being put together as an afterthought, are either rather unspecific and vague, highly specific but of questionable relevance, or implausibly ambitious, and hence fairly meaningless. Crucially, most will say *what* should be learned and perhaps *how*, but not necessarily *why*, i.e. they neglect to explain the relevance. But *why* lies at the centre of education and a key driver of motivation, which is why the IMiLI resources strive to explain why the topics taught are important, relevant or simply of high interest.

We are amazed how resource creators have managed to crystalize out in enchanting stories the key messages of the microbiology of familiar everyday processes or exciting exploration science that will undoubtedly be exciting to children. They have discovered how to relate their topics to children and, in the process, learned to explain their work to their own children.

***Willy Verstraete***

The world of learning is now dominated by 'regulators'. The latter have to make sure that the pupils reach certain 'minimal criteria' which are subject to terrible discussions between various stakeholders, such as the STEM and Greek-Latin scholars. But youngsters enjoy hands-on practical exercises and are eager to see the results. “*Ora et labora”*, the slogan of the Benedictines, emphasizes the need for physical activity in addition to mental activity and learning (<https://en.wikipedia.org/wiki/Ora_et_labora>): work with the hands and not only on keyboards! Hence: it can be a *unique selling point* if the IMiLI focusses on a variety of hands-on examples from daily life and environmental well-being. The IMiLI bringing a chance for youngsters to break out of the 'sterile bubble ' in which they tend to exist today is worth emphasizing.

REFERENCES

Canfield, D.E., Glazer, A.N. & Falkowski, P.G. (2010) The evolution and future of Earth's nitrogen cycle. Science, 330, 192–196.

O'Neill, J. (2016) Tackling drug‐resistant infections globally: final report and recommendations. <https://amr-review.org/sites/default/files/160525_Final%20paper_with%20cover.pdf>

Rockström, J., Steffen, W., Noone, K., Persson, A., Stuart Chapin, F., III, Lambin, E.F. et al. (2009) A safe operating space for humanity. Nature, 461, 472–475. Available from: <https://doi.org/10.1038/461472a>

Rucker, H. (2023) Enigmatic evolution of microbial nitrogen fixation: insights from Earth's past. Trends in Microbiology. Available from: https://doi.org/10.1016/j.tim.2023.03.011

Taylor, P. & Reeder, R. (2020) Antibiotic use on crops in low and middle‐income countries based on recommendations made by agricultural advisors. CABI Agriculture and Bioscience, 1. Available from: https://doi.org/10.1186/s43170-020-00001-y
